# Expression of a *Colletotrichum* polyketide synthase gene in *Aspergillus nidulans* leads to unexpected conjugates with a host metabolite

**DOI:** 10.1007/s00203-025-04258-7

**Published:** 2025-02-06

**Authors:** David Breyer, Leyao Chen, Jenny Zhou, Zhang-Hai Li, Shu-Ming Li

**Affiliations:** Institut für Pharmazeutische Biologie und Biotechnologie, Fachbereich Pharmazie, Philipps-Universität Marburg, Robert-Koch Straße 4, 35037 Marburg, Germany

**Keywords:** Phytopathogenic fungus, *Colletotrichum higginsianum*, *Aspergillus nidulans*, Higginidulans A–B, Heterologous expression, Crosstalk reactions

## Abstract

Heterologous expression of the putative 1,3,6,8-tetrahydroxynaphthalene synthase gene *ChPKS* from *Colletotrichum higginsianum* in *Aspergillus nidulans* led to the formation of at least eight new compounds. LC-MS analysis proved them as coupling products of 1,3,6,8-tetrahydroxynaphthalene with an intermediate of the cichorine biosynthetic pathway. Comprehensive NMR analysis confirmed the structures of the two predominant products higginidulans A and B. Deletion of the backbone gene of the cichorine pathway in host strain *Aspergillus nidulans* abolished the formation of higginidulans. Heterologous expression of *ChPKS* in the alternative *Penicillium crustosum* expression host resulted in the formation of the expected product 1,3,6,8-tetrahydroxynaphthalene, which was confirmed by acetylation and structural elucidation. This study provides an additional example of unexpected natural product formation by crosstalk of biosynthetic pathways derived from different species. Moreover, it highlights the importance of using alternative host systems for gene expression.

**Supplementary Information:**

The online version contains supplementary material available at 10.1007/s00203-025-04258-7.

## Introduction

**Fig. 1:**
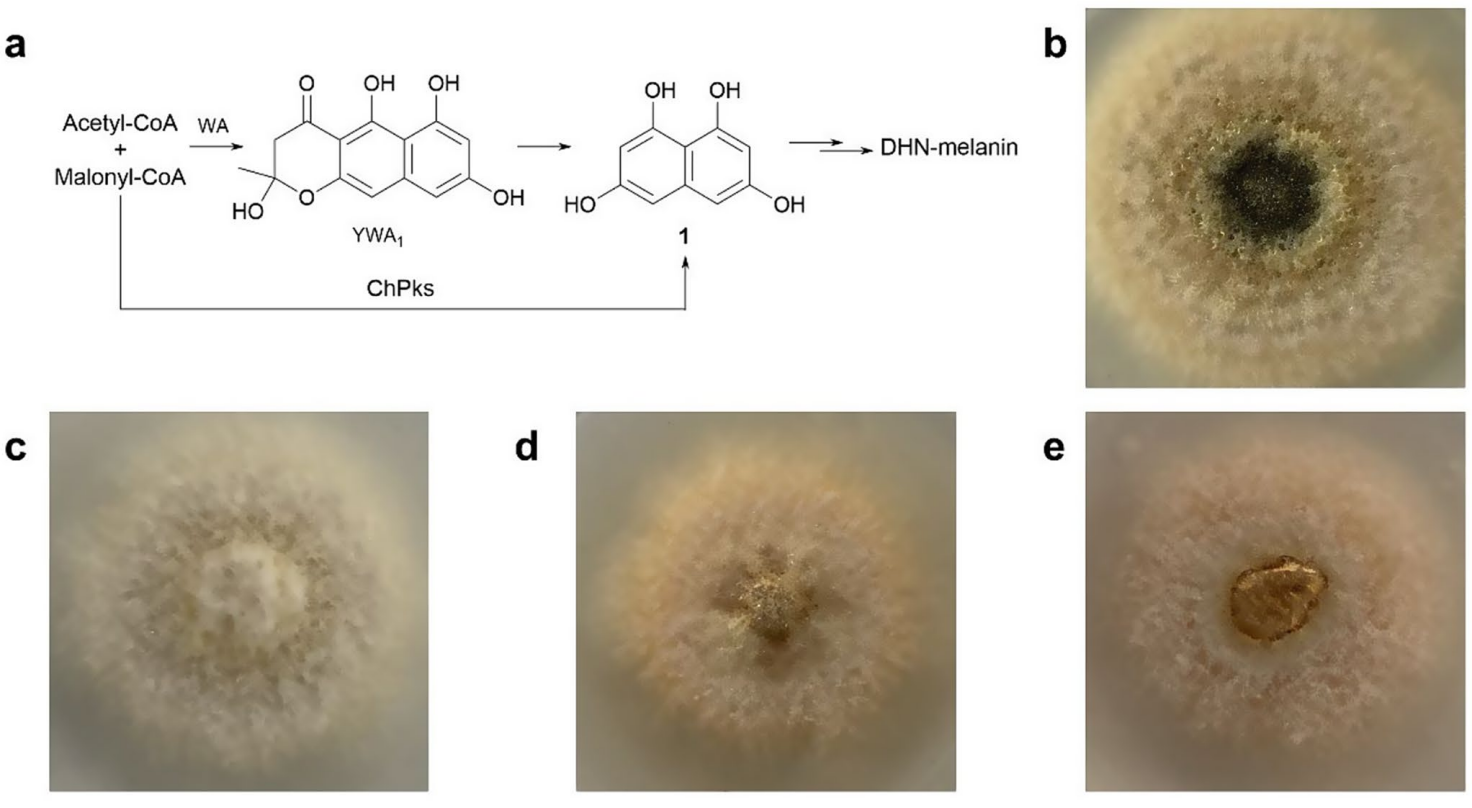
DHN-melanin as spore pigment. (**a**) Formation in fungi via YWA1 and 1,3,6,8-THN (**1**), Phenotypes of (**b**) *A. nidulans* LO8030 (wild type), (**c**) *A. nidulans* SSt01 (*wA-PKS*::*PgpdA-AfpyrG* in LO8030), (**d**) *A. nidulans* DB04 (*wA‐PKS*::*PgpdA-CH35J_010369-AfpyrG* in LO8030), (**e**) *A. nidulans* DB09 (ΔANIA_06448::*Afpyro in* DB04)

The genus *Colletotrichum* belongs to the fungal family of *Glomerellaceae* and is known for its plant pathogenicity (Dean et al. 2012). The pigment melanin plays an important role in this genus, offering protection against environmental stressors and thus ensuring survival. The absence of the melanin gene in *Colletotrichum* strains has been shown to result in a delayed penetration of plant tissue, thereby affecting their plant pathogenicity (Wang et al. 2021). A number of studies have demonstrated that *Colletotrichum* species primarily produce dihydroxynaphthalene-melanin (DHN-melanin) (Wang et al. 2021; Duan et al. 2023). At least two different biosynthetic pathways have been reported for the formation of the important DHN-melanin precursor 1,3,6,8-tetrahydroxynaphthalene (1,3,6,8-THN) (Liang et al. 2022). While polyketide synthases (PKSs) from *Colletotrichum* strains are capable of directly producing 1,3,6,8-THN, many *Aspergillus* and *Penicillium* species synthesize a heptaketide, such as the naphthopyrone YWA1, as its precursor (Tsai et al. 2001; Schumacher 2016; Perez-Cuesta et al. 2020). Subsequent oxidation and dehydration result in the formation of 1,8-dihydroxynaphthalene (1,8-DHN) and its polymerization product, DHN-melanin (Fig. 1a) (Eisenman and Casadevall 2012; Shafreen et al. 2024).

The ascomycete fungus *Colletotrichum higginsianum* MAFF 305635 was firstly isolated from *Brassica rapa* in Japan in 1980 (Horie et al. 1988). It is the causative agent of anthracnose disease in a diverse range of cruciferous plants. *C. higginsianum* has the potential to infest almost all parts of the plants, which renders it a significant economic concern (Damm et al. 2014). In addition to its plant pathogenicity, the *Colletotrichum* genus was found to exhibit a considerable secondary metabolite spectrum, indicating a promising opportunity for the discovery of new compounds (Moraga et al. 2019). For this reason, the objective of this study was to establish a protocol for the heterologous expression of biosynthetic genes and gene clusters from *C. higginsianum* in commonly used host strains, such as *Aspergillus nidulans* LO8030 (Chiang et al. 2013). In analogy to a previous work of our group, we also chosen a gene for pigment formation, namely the *ChPKS* gene for the biosynthesis of the DHN-melanin precursor 1,3,6,8-THN (Kindinger et al. 2019). We expected the recovery of the green conidial pigment after a successful heterologous expression of the *ChPKS* gene.

## Materials and methods

### Sequence analysis

Biosynthetic gene clusters were predicted by using antiSMASH (https://antismash.secondarymetabolites.org). Intron and exon sequences were analyzed by 2ndFind (https://biosyn.nih.go.jp/2ndfind/). Enzyme functions were predicted by using the NCBI BLAST online bioinformatic tool (https://blast.ncbi.nlm.nih.gov/Blast.cgi). Domain structure analysis was carried out by using InterproScan (https://www.ebi.ac.uk/interpro/).

### Strains and culture conditions

*Escherichia coli* DH5α and *Saccharomyces cerevisiae* HOD114-2B (Table S1) were cultivated as described in previous protocols (Xiang and Li 2022; Zhou et al. 2024).

For spore production, *C. higginsianum* MAFF 305635 (Table S1) was cultivated on oatmeal agar (60 g/L oat bran (Rossmann, Burgwedel, Germany), 0.1% yeast extract, 1.27% agar) at 25 °C for 21 days.

*A. nidulans* and *Penicillium crustosum* strains (Table S1) were cultivated on glucose minimal medium (1.1 g/L glucose monohydrate, 50 mL/L salt solution, 1 mL/L trace elements and 0.5% yeast extract, 1.6% agar) with the required supplements (1 g/L uracil, 1.2 g/L uridine, 2.5 mg/L riboflavine, or 0.5 g/L pyridoxine) at 37 °C–25 °C, as described in previous protocols (Zheng et al. 2020; Zhou et al. 2024).

### Isolation of genomic DNA

For the isolation of genomic DNA, fungal strains were cultivated in potato dextrose broth (24 g/L potato dextrose broth, Carl Roth, Karlsruhe, Germany) or liquid minimal medium (1.1 g/L glucose monohydrate, 50 mL/L salt solution, 1 mL/L trace elements and 0.5% yeast extract) for 3 days at 37 °C for *Aspergillus* and 25 °C for *Penicillium* and *Colletotrichum* strains. Isolation of genomic DNA was performed as described in a previous protocol (Xiang et al. 2022).

### PCR amplification, cloning, plasmid construction, and fungal transformation

Plasmids and primers constructed and used in this study are given in Tables S2 and S3. Primers were synthesized by Seqlab GmbH (Göttingen, Germany). Phusion High-Fidelity DNA polymerase from New England Biolabs (Ipswich, Massachusetts) was used for DNA amplification by polymerase chain reaction (PCR).

To obtain pDB04 for heterologous expression, the entire *ChPKS* (CH35J_010369) gene of 6755 bp was obtained in two fragments overlapping by 292 bp. The two primer pairs DB24/DB25 and DB26/DB27 (Table S3) and *C. higginsianum* MAFF305635 genomic DNA as template were used for the amplification of fragments 1 and 2, respectively. PCRs were conducted in a T100 thermal cycler (Bio-Rad Laboratories, Hercules, California) following the manufactural protocol. The two fragments were then assembled by a 34–35 bp overlap to the SfoI-linearized vector pSSt05 (Stierle and Li 2022) (Table S2) via homologous recombination in *S. cerevisiae* HOD114-2B (Table S1), yielding pDB04 (Yin et al. 2013). In this way, the PKS gene *ChPKS* was inserted between the *PgpdA* promoter and the *AfpyrG* marker.

To generate the constructs pZL175 and pZL176 for the deletion of *pkbA* (ANIA_06448) in the cichorine pathway (Table S2), approximately 1000 bp fragments of the 5’-UTR (untranslated region) and 3’‐UTR of *pkbA* were amplified by PCR from genomic DNA of *A. nidulans* LO8030 using the primer pairs ZL_pZL175_REV/ZL_pZL175_FOR and ZL_pZL176_REV/ZL_pZL176_FOR (Table S3). Vector pYWB1 (Janzen et al. 2023b) (Table S2) was linearized by NheI/HindIII and NotI, respectively. The fragments and linearized vectors were assembled in *E. coli* DH5α to give pZL175 and pZL176.

Germination, protoplastation, and transformation of *Aspergillus* and *Penicillium* strains, as well as preparation of the associated media and solutions were carried out and prepared as described in previous protocols (Zhou and Li 2021; Janzen et al. 2023a; Zhou et al. 2024). PCR amplification (Figs. S1 and S2) was used to confirm transformants *A. nidulans* DB04 (*ChPKS* expression), *A. nidulans* DB09 (*pkbA* deletion), and *P. crustosum* JZ58 (*ChPKS* expression). The transformants were then cultivated on rice medium (20 g royal tiger rice with 30 mL H_2_O) for 21 days with the required supplements. The ethyl acetate extracts were analyzed by liquid chromatography-mass spectrometry (LC-MS).

### LC-MS analysis

**Fig. 2:**
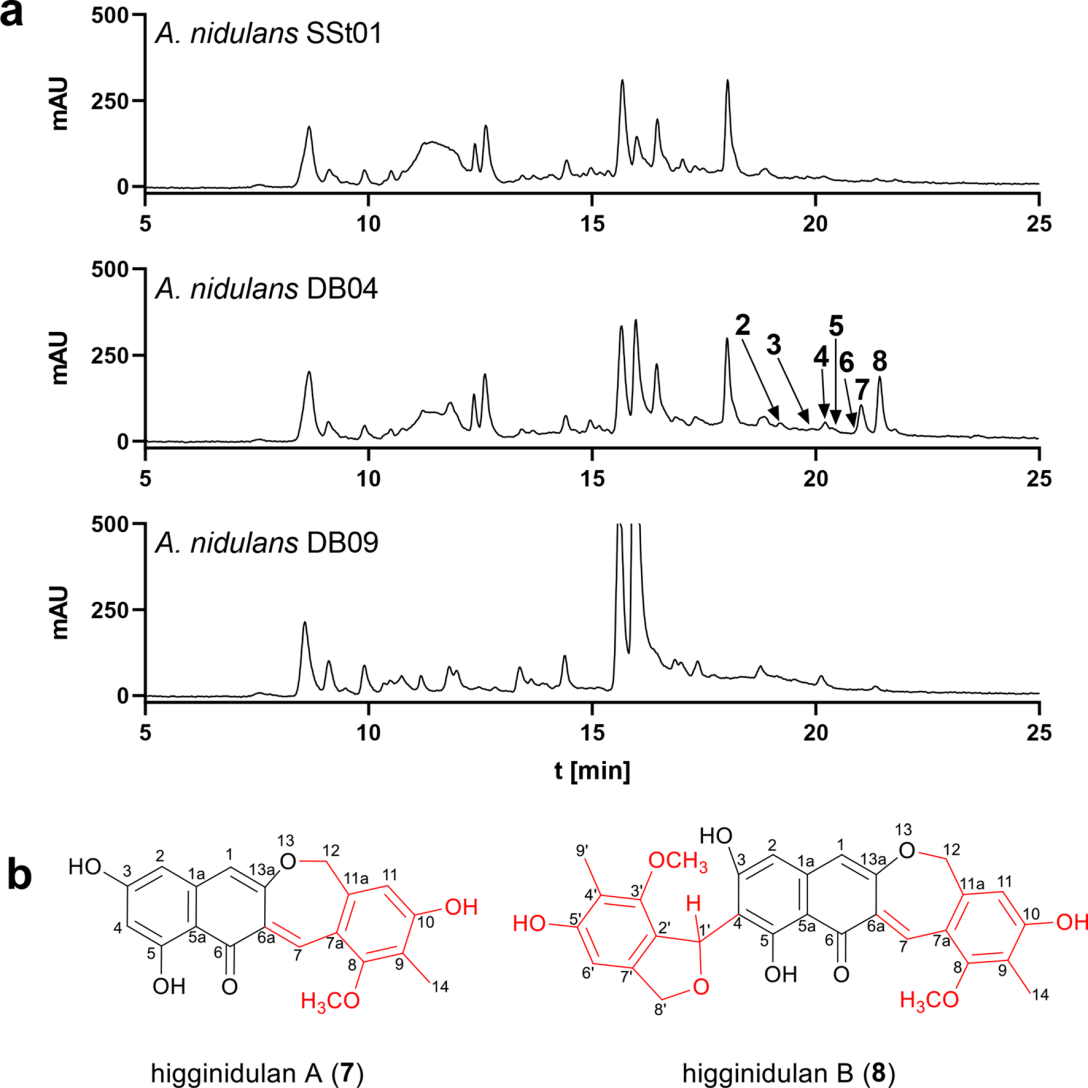
(**a**) LC-MS analysis of culture extracts from *A. nidulans* SSt01 (control), DB04 and DB09 after 21 days. UV absorptions at 283 nm are illustrated. (**b**) Structures of higginidulans A (**7**) and B (**8**)

Secondary metabolite analysis was carried out on a micrOTOF-QIII mass spectrometer (Bruker, Bremen, Germany), which is connected to an Agilent 1260 HPLC system (Agilent Technologies, Böblingen, Germany) equipped with a VDSpher PUR 100 C18-M-SE (150 × 2 mm, 3 μm) column (VDS optilab Chromatographietechnik GmbH, Berlin, Germany). The mobile phase with a flow rate of 0.30 mL/min is composed of H_2_O and acetonitrile, both containing 0.1% formic acid. The elution is carried out via a linear gradient from 5 to 100% acetonitrile within 30 min. The data obtained were analyzed with the Compass DataAnalysis 4.2 software (Bruker, Bremen, Germany). UV detections and extracted ion chromatograms (EICs) are shown in Figs. 2 and 4, S3, and S4.

### Large-scale fermentation, extraction, and isolation of secondary metabolites

*A. nidulans* strain DB04 was cultivated in ten flasks each containing 100 g rice, 150 mL H_2_O and the required supplements at 25 °C for 22 days. The ethyl acetate extract (5.3 g) was fractionated on a silica gel column by using a gradient of dichloromethane and methanol in a ratio ranging from 50:1 to 0:100.

A semi-preparative Agilent 1200 HPLC system equipped with an Agilent Eclipse XDB-C18 (9.4 × 250 mm, 5 μm) column (Agilent Technologies, Böblingen, Germany) was then used to isolate compounds **7** and **8** from the obtained fractions. 12.0 mg of **7** were obtained by isocratic elution with water (A) and acetonitrile (B) in a ratio of 45:55. 8.0 mg of **8** were isolated using a linear gradient from 45 to 55% B within 30 min.

Transformant *P. crustosum* JZ58 was cultivated in eight flasks with rice medium at 25 °C for 10 days. The crude extract (10.5 g) was immediately acetylated by adding acetic anhydride (170 mmol) and sodium acetate· (2.4 mmol) for 24 h at room temperature. The mixture was extracted with 120 mL ethyl acetate and washed with 120 mL of a saturated solution of sodium bicarbonate for three times. After evaporation of the solvent, the acetylated extract was applied to a preparative Agilent 1260 Infinity II HPLC system, equipped with an Agilent Eclipse VDSpher PUR 100C18-M-SE (20 × 150 mm, 5 μm) column (Agilent Technologies, Böblingen, Germany). 7.8 mg of **11** were isolated and subjected to MS and NMR analyses.

### NMR analysis and structural elucidation

NMR spectra were recorded on a JEOL ECA-500 MHz spectrometer (JEOL ltd., Tokyo, Japan). The samples were dissolved in DMSO-*d*_*6*_, acetone-*d*_*6*_, or CDCl_3_. The spectra were analyzed with MestReNova 9.0.0 (MestreNova, Santiago de Compostela, Spain).

### Physicochemical properties of the compounds described in this study

#### Higginidulan A (7)

dark red powder; ^1^H- and ^13^C-NMR data are given in Table S4; HRMS (ESI-TOF) *m/z*: [M + H]^+^ Calculated for C_20_H_16_O_6_ 353.1020; Found 353.1039 (Fig. S3).

#### Higginidulan B (8)

dark red powder; ^1^H- and ^13^C-NMR data are given in Table S5; HRMS (ESI-TOF) *m/z*: [M + H]^+^ Calculated for C_30_H_26_O_9_ 531.1650; Found 531.1679 (Fig. S3).

#### 1,3,6,8-tetraacetoxynaphthalene (11)

black powder; the ^1^H-NMR spectrum is shown in Fig. S14; HRMS (ESI-TOF) *m/z*: [M + H]^+^ Calculated for C_18_H_16_O_8_ 361.0918; Found 361.0949 (Fig. 4a).

### Availability of the genome sequences

The genome sequences of *C. higginsianum* IMI 349063 and MAFF305635 are available in NCBI/Genbank under the accession numbers GCA_001672515.1 and GCA_004920355.1, respectively.

## Results and discussion

### Bioinformatic analysis of *ChPKS* genes

To prove that the commonly used *A. nidulans* LO8030 host is able to successfully express genes from the genus *Colletotrichum*, the gene coding for the PKS TIC92505 from *C. higginsianum* MAFF 305635 was selected for heterologous expression. TIC92505 exhibits an identical amino acid sequence to that of the known ChPKS (XP_018155915) from *C. higginsianum* IMI 349063, which was demonstrated to be involved in the DHN-melanin biosynthesis (Duan et al. 2023). Accordingly, TIC92505 was also designated as ChPKS. BLASTp analysis revealed that ChPKS shares a sequence identity of 91% on the amino acid level with the PKS1 (BAA18956) from *C. lagenarium* 104-T. In previous studies, PKS1 was demonstrated to catalyze the formation of the DHN-melanin precursor 1,3,6,8-THN (**1**) (Takano et al. 1995; Damm et al. 2013). It was therefore proposed that the identified ChPKS from *C. higginsianum* MAFF 305635 is involved in melanin pigment formation. Prediction by using 2ndFind indicated that *ChPKS* with a length of 6755 bp comprises four exons with lengths of 45, 5913, 292, and 293 bp, interrupted by three intron sequences with 57, 80, and 75 bp, respectively.

### Heterologous expression of *ChPKS* gene in *Aspergillus nidulans* LO8030

Given its involvement in pigment formation, the successful expression of *ChPKS* in *A. nidulans* can be easily observed by a change in the phenotype. The *ChPKS* gene from *C. higginsianum* MAFF 305635 (Table S1) was amplified by PCR and assembled into the vector pSSt05 (Table S2) by homologous recombination in *S. cerevisiae* HOD114-2B as described previously (Yin et al. 2013). The obtained plasmid pDB04, with *ChPKS* under the control of the *gpdA* promoter (Table S2), was introduced into *A. nidulans* LO8030 by polyethylene-glycol-mediated protoplast transformation (Chiang et al. 2013; Yin et al. 2013). The resulting transformant *A. nidulans* DB04 (Table S1) was confirmed by PCR verification (Fig. S1a). Successful expression of pDB04 at the *A. nidulans wA* integration site results in the replacement of the *wA* gene by *ChPKS*. Previous studies demonstrated that the *wA* gene codes for a PKS responsible for the formation of YWA1. The naphthopyrone YWA1 is subsequently converted to 1,3,6,8-THN (**1**) as part of the DHN-melanin pathway (Fig. 1a) (Gao et al. 2022).

Additionally, the expression of the *PKS1* gene from *C. lagenarium* 104-T in host strain *A. oryzae* was shown to result in the formation of 1,3,6,8-THN (**1**) (Fujii et al. 1999). Given the high degree of homology between *ChPKS* and the *PKS1* gene, it was expected that the heterologous expression of *ChPKS* in *A. nidulans* would result in the biosynthesis of **1** and the formation of colored spores (Fetzner et al. 2014). Unexpectedly, transformant DB04 was unable to restore the green spore pigmentation characteristic of the wild-type strain LO8030 or the dark brown spore pigments typically associated with DHN-melanin alone (Thines et al. 1995). Instead, it exhibited a pale rose coloration (Fig. 1d). This change in the phenotype indicated that the *wA* gene had been replaced, yet the resulting 1,3,6,8-THN (**1**) was not effectively converted to DHN-melanin in DB04. It can be speculated that the formed 1,3,6,8-THN (**1**) was further metabolized to other products.

### Higginidulans A and B production and structure elucidation

To prove the hypothesis of further metabolization of 1,3,6,8-THN (**1**), *A. nidulans* DB04 was cultivated in rice medium supplemented with riboflavin and pyridoxin at 25 °C for 21 days. In parallel, control strain *A. nidulans* SSt01 (Table S1), which harbours the empty vector pSSt05, was grown under the same conditions. LC-MS analysis of the ethyl acetate extracts revealed the presence of at least seven additional peaks **2**−**8** in DB04, in comparison to the control strain SSt01 (Fig. 2a and S3). The [M + H]^+^ ions were detected at *m/z* 353.104 ± 0.005 for **2** and **7**, *m/*z 339.0848 for **3**, *m/z* 531.168 ± 0.005 for **4**, **8**, and **6**, and *m/*z 517.1491 for **5**, respectively. Compounds **7** and **8** were found to be the predominant products, accounting for nearly 61% of the total products. However, the ChPKS product **1** was not identified in the culture extracts under the used conditions.

To elucidate the structures of **2**–**8** and their biosynthetic origin, *A. nidulans* DB04 was subjected to large-scale fermentation. The predominant compounds **7** and **8** were isolated from a 22-day old rice culture of DB04 and subjected to NMR analysis (Tables S4 and S5, Figs. S5 – S13).

Compound **7** was obtained as a dark red powder. Based on its [M + H]^+^ ion at *m/z* 353.1039 in the positive HRESIMS, the molecular formula was deduced to be C_20_H_16_O_6_, implying 13 degrees of unsaturation. The 20 carbon resonances in the ^13^C-NMR spectrum of **7** (Fig. S6) comprise a carbonyl carbon at *δ*_C_ 186.1 ppm, five sp^2^ oxygenated tertiary carbons at *δ*_C_ 166.1, 165.3, 161.8, 161.8, and 155.5 ppm, six sp^2^ tertiary carbons at *δ*_C_ 141.0, 140.5, 126.5, 119.3, 118.0, and 106.6 ppm, five sp^2^ methines at *δ*_C_ 136.8, 110.8, 106.3, 104.0, and 99.5 ppm, an sp^3^ oxygenated methylene at *δ*_C_ 71.9 ppm, an *O*-methyl carbon at *δ*_C_ 62.3 ppm, and an sp^3^ methyl carbon at *δ*_C_ 8.9 ppm. The ^1^H-NMR spectrum in DMSO-*d*_*6*_ showed two uncoupling olefinic protons at *δ*_H_ 8.42 (H-7) and 6.73 (H-11) ppm, three coupling olefinic protons at *δ*_H_ 6.26 (d, *J* = 2.2 Hz, H-2), 6.10 (dd, *J* = 2.2, 1.4 Hz, H-1) and 6.05 (t, *J* = 2.2 Hz, H-4) ppm, an *O*-methylene group at *δ*_H_ 4.88 ppm (s, H-12), an *O*-methoxy group at *δ*_H_ 3.76 ppm (s, 8-OCH_3_), and a methoxy group at *δ*_H_ 2.09 ppm (s, H-14).

**Scheme 1:**
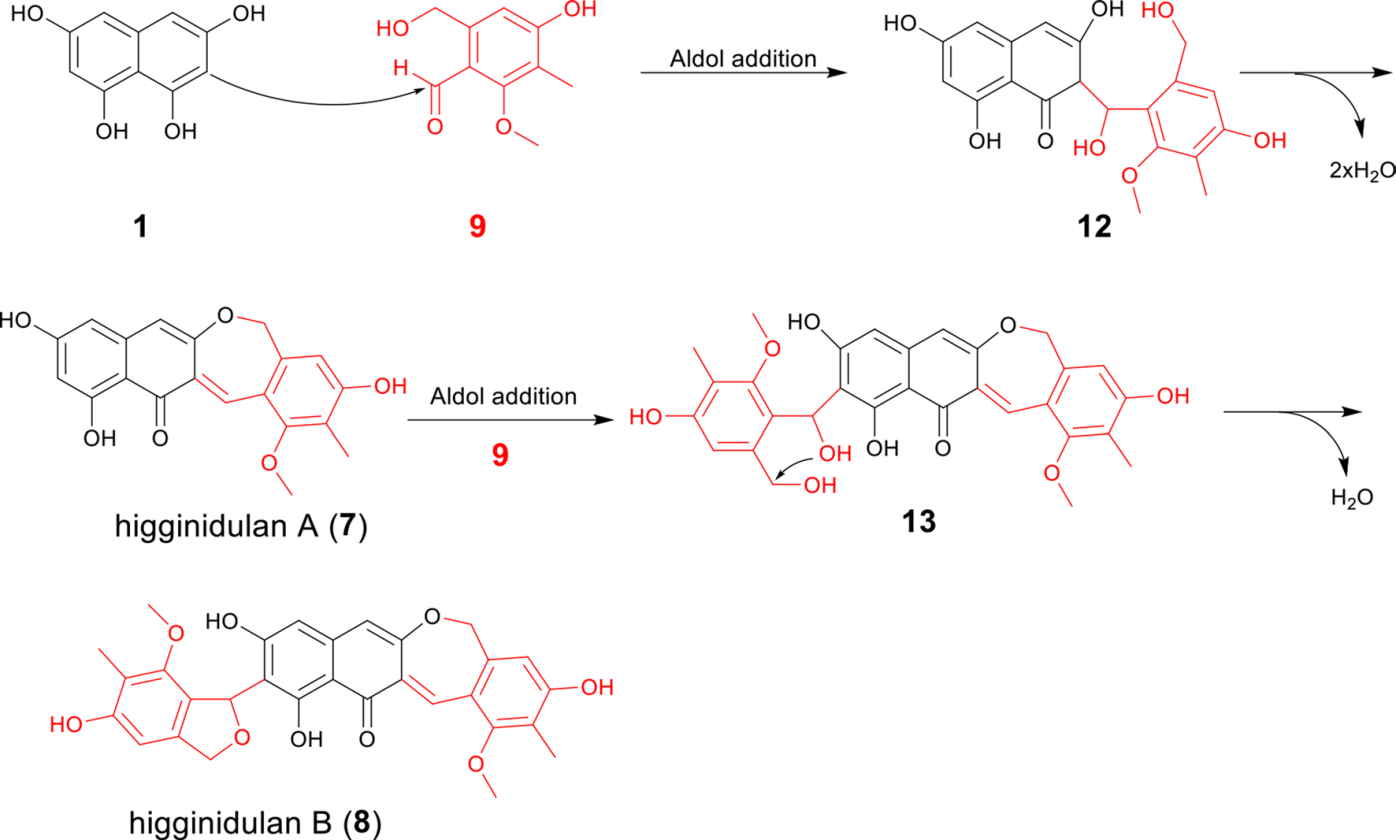
Proposed coupling reactions between **1** and **9** to form higginidulans A (**7**) and B (**8**)

The HMBC correlations from H-1 to C-1a, C-2, C-5, C-5a, C-6a and C-13a, from H-2 to C-3, C-4, C-5a and C-6, and from H-4 to C-3, C-5, C-5a and C-6 confirmed the presence of the trihydroxy-1(2*H*)-naphthalenone moiety. The HMBC correlations from H-11 to C-7, C-7a, C-9, C-10, C-12 and C-14 prove the presence of a *penta*-substituted phenyl unit. Detailed analysis of the HMBC correlations from H-7 to C-6, C-8, C-11a and C-13a, as well as from H-12 to C-7a and C-13a, confirmed the connection between the two units via an ether bond between C-12 and C-13a and a double bond between 6a and 7, indicating the presence of a seven-membered dihydrooxepin ring. Furthermore, the NOESY correlation between OCH_3_ at *δ*_H_ 3.76 ppm and the uncoupling olefinic proton at *δ*_H_ 8.42 ppm confirmed the position of OCH_3_ at C-8. Thus, the structure of **7** (Fig. 2b) can be considered as a coupling product of 1,3,6,8-THN (**1**) and the aldehyde **9** (Scheme 1), which has not been described before. Compound **7** was termed higginidulan A hereafter.

Compound **8** was also obtained as a dark red powder. Its molecular formula of C_30_H_26_O_9,_ deduced from its [M + H]^+^ ion at *m/z* 531.1679 in the positive HRESIMS, implies 18 degrees of unsaturation. The ^13^C NMR spectrum of **8** (Fig. S11) showed 30 carbon resonances comprising a carbonyl carbon (*δ*_C_ 188.2), seven sp^2^ oxygenated tertiary carbons at *δ*_C_ 167.3, 166.4, 164.6, 163.2, 157.4, 156.9, and 154.6 ppm, ten sp^2^ tertiary carbons at *δ*_C_ 141.7, 141.3, 140.8, 128.4, 124.7, 121.2, 119.3, 116.7, 113.0, and 108.0 ppm, five sp^2^ methines at *δ*_C_ 137.9, 111.6, 107.1, 104.9, and 103.4 ppm, two sp^3^ oxygenated methylenes at *δ*_C_ 74.0 and 73.1 ppm, an sp^3^ oxygenated methine at *δ*_C_ 76.6 ppm, two *O*-methyl carbons at *δ*_C_ 63.0 and 60.3 ppm, and two sp^3^ methyl carbons at *δ*_C_ 9.2 and 9.0 ppm. The ^13^C NMR data of **8** differ from those of **7** by an additional aromatic unit in **8**. The downfield chemical shift of C-4 at *δ*_C_ 113.0 with a Δ*δ*_C_ 13.5 ppm in comparison to that of **7** and the HMBC correlation from H-1’ to C-3, C-4 and C-5 indicated that the additional aromatic unit must be located at C-4. The NMR data of the additional aromatic unit are very similar to those of the aldehyde **9** (Grau et al. 2019) with the exception for C-1’ at *δ*_C_ 76.6 ppm. These data proved that a substituted dihydrobenzofuran derived from **9** is attached to **7** via a C4-C1’ bond. Thus, the structure of **8** was established as a coupling product of one molecule of 1,3,6,8-THN (**1**) and two molecules of **9** (Scheme 1), and termed higginidulan B (Fig. 2b).

### Deletion of *pkbA* gene in *Aspergillus nidulans* DB04

Based on the high homology of ChPKS to known 1,3,6,8-THN synthases as mentioned above, the additional structural moieties in **7** and **8** are very likely derived from the host strain *A. nidulans* LO8030. A recent study demonstrated the involvement of a cichorine pathway product from *A. nidulans* in the formation of hybrid molecules after heterologous expression (Sanchez et al. 2012; Lu et al. 2024). As mentioned above, the aldehyde **9** can also be considered as a cichorine precursor. To prove this hypothesis, the backbone gene *pkbA* (Sanchez et al. 2012), coding for a nonreducing PKS as part of the cichorine biosynthetic pathway, was deleted in *A. nidulans ChPKS* expression strain DB04. The resulting Δ*pkbA* mutant *A. nidulans* DB09 (Table S1) was confirmed by verification via PCR (Fig. S2a) and cultivated on rice medium supplemented with riboflavin under light exclusion at 25 °C. LC-MS analysis of the 21-day old culture extracts revealed the abolishment of **7** and **8** in DB09 (Fig. 2a), thereby confirming the involvement of the cichorine cluster in their formation. It was expected that the elimination of the cichorine precursor **9** may increase the availability of **1** for the formation of DHN-melanin and therefore restore spore pigment formation. However, the Δ*pkbA* strain DB09 was still unable to produce spore pigments and exhibited a phenotype similar to that observed in *A. nidulans ChPKS* expression strain DB04 (Fig. 1d) (Thines et al. 1995). Furthermore, for unknown reasons, LC-MS analysis of the culture extracts did not lead to detection of **1** or its oxidation products in *A. nidulans* DB09 (Fig. S4) (Fujii et al. 1999; Fetzner et al. 2014). This raised the question about the function of *ChPKS.*

### Heterologous expression of *ChPKS* gene in *Penicillium crustosum* JZ52

**Fig. 3:**
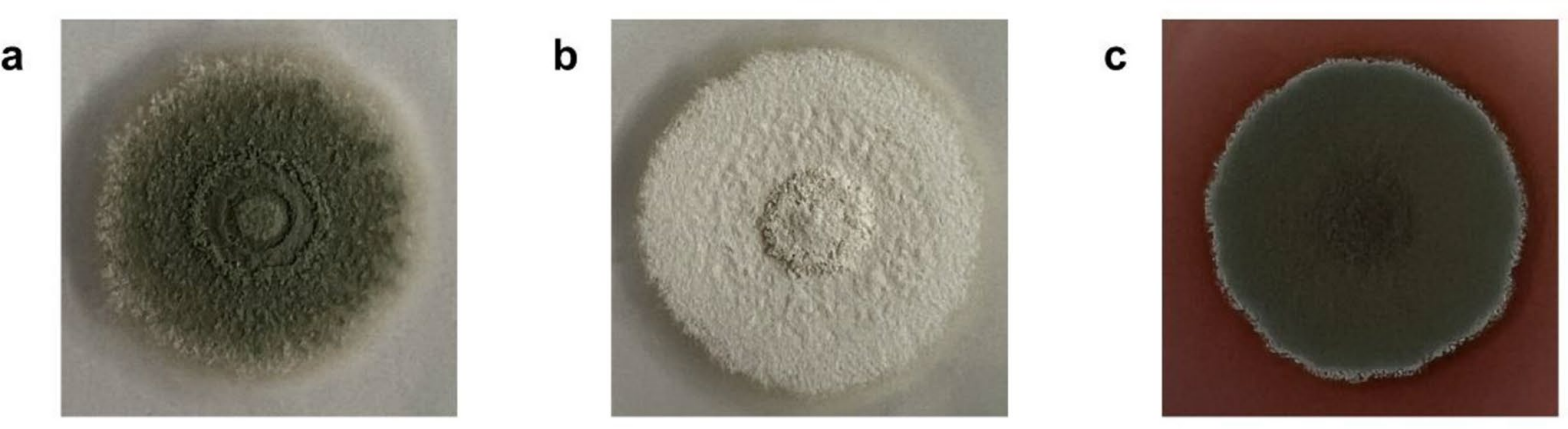
(**a**) *P. crustosum* JZ52 (*Δpcr4401*::*wA-PKS Δpcribo ΔpyrG*), (**b**) *P. crustosum* JZ57 (*wA-PKS*::*PgpdA-AfpyrG* in JZ52) and (**c**) *P. crustosum* JZ58 (*wA‐PKS*::*PgpdA-CH35J_010369-AfpyrG* in JZ52). The strains grown on GMM agar plates with appropriate supplementation for 7 days at 25–37 °C. The production of 1,3,6,8-THN (**1**) by JZ58 resulted in an intense red pigmentation of the surrounding medium

To prove the function of *ChPKS*, the alternative host system *P. crustosum* JZ52 was selected for the heterologous expression of this gene (Zhou et al. 2024). Polyethylene-glycol-mediated transformation of JZ52 with pSSt05 and pDB04 (Table S2) resulted in transformants *P. crustosum* JZ57 and JZ58 (Table S1), respectively. While empty vector control JZ57 exhibited an albino phenotype (Fig. 3b), *ChPKS* expression strain JZ58 produced colored spores and even colored the surrounding agar medium red, indicating the formation of 1,3,6,8-THN (**1**) (Fig. 3c) (Fetzner et al. 2014).

**Fig. 4:**
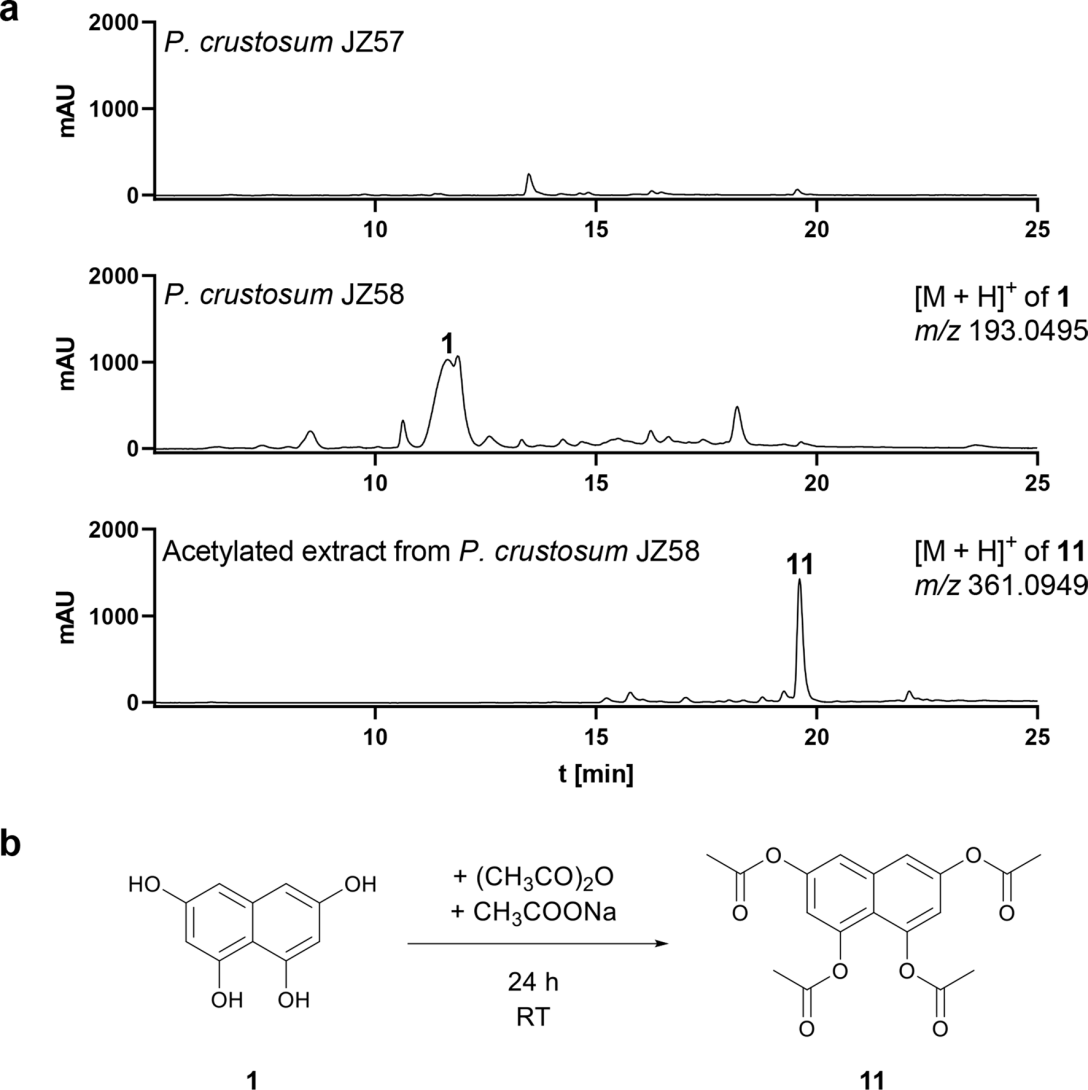
(**a**) LC-MS analysis of culture extracts from *P. crustosum* JZ57 (control) and JZ58 with compound 1 after 21 days, as well as the acetylated extract from JZ58 after 10 days. UV absorptions at 283 nm are illustrated. (**b**) Conversion of 1 to 11 by acetylation

After confirmation by PCR (Figs. S1a and S1b), both strains were cultivated on rice medium supplemented with riboflavin under light exclusion at 25 °C. Samples were taken after 21 days, extracted with ethyl acetate and then measured with LC-MS. As shown in Fig. 4a, a dominant peak was detected for the *ChPKS* expression mutant *P. crustosum* JZ58, which was not present in control strain JZ57. The observed [M + H]^+^ ion at *m/z* 193.0495 corresponds to that of the expected 1,3,6,8-THN (**1**) (Zhang et al. 2017).

### 1,3,6,8-THN production and structure elucidation

A large-scale fermentation was carried out to isolate **1** from 10-day old cultures of JZ58. After unsuccessful isolation of **1** due to its sensitivity to oxidation (Fujii et al. 1999), we decided to obtain it in its acetylated form through acetylation of the crude extract. The isolated tetraacetylated product of 1,3,6,8-THN (**1**), 1,3,6,8-tetraacetoxynaphthalene (**11**), was subjected to ^1^H-NMR analysis, thereby confirming the deduced structure of **1** (Fig. 4b and S14) (Fujii et al. 1999). These data indicate that the alternative host system *P. crustosum* JZ52, like *A. nidulans*, is capable of expressing genes from *C. higginsianum*. Moreover, the function of *wA* in *P. crustosum* can be restored by *ChPKS* via **1**.

### Proposed mechanism for coupling of 1,3,6,8-THN with cichorine intermediate

Due to the low quantities, the other products could not be obtained in substantial amounts from *A. nidulans* DB04 for structural elucidation by NMR analysis. However, the LC-MS data indicated that they are very likely also adducts of **1** with **9** or its demethylated derivatives via different coupling patterns (Fig. S3). In comparison to **7** and **8**, compounds **3** and **5** exhibited a mass decrease of 14 Da, thereby suggesting the absence of a methyl group in both cases. A previous study indicated that the reaction order within the cichorine biosynthetic pathway, in particular the methylation of the phenolic hydroxy groups, remains unresolved (Sanchez et al. 2012; Zhao et al. 2023). Consequently, the coupling reaction with **1** can occur with both methylated and nonmethylated cichorine precursors. The detection of **4** and **6** as isomers of **8** was based on the observation of nearly identical molecular masses. Additionally, LC-MS analysis revealed that compound **2** is an isomer of **7**. The existence of the isomers can be attributed to the fact that **1** presents multiple targets for addition reactions (Fig. S3). Based on our findings, we propose the following mechanism for the formation of higginidulans A (**7**) and B (**8**) (Scheme 1). The reaction between **1** and **9** leads to the formation of compound **12** via an aldol addition. Elimination of two water molecules results in the formation of the dihydrooxepin ring in higginidulan A (**7**). Attachment of a second molecule of **9** to **7** results in the formation of intermediate **13**, which undergoes water elimination to form the dihydrobenzofuran ring in higginidulan B (**8**).

A number of hybrid products with one or two cichorine moieties have been identified in *A. nidulans*, including aspercryptin B1 and andicichorines A–C (Chiang et al. 2016; Zhao et al. 2023). In the case of the heterologously expressed restrictin biosynthetic gene cluster, a coupling reaction was observed between the primary amine of the restrictin glycyl residue and the aldehyde of a cichorine intermediate derived from *A. nidulans* (Lu et al. 2024). In the present study, one and two molecules of a cichorine precursor were connected with the ChPKS product 1,3,6,8-THN (**1**) by C-C bond(s) via aldol addition. This resulted in the formation of pathway crosstalk products, including higginidulans A (**7**) and B (**8**), which contain dihydrooxepin and dihydrobenzofuran ring systems. To the best of our knowledge, such cichorine derivatives have not been reported previously.

## Conclusion

In summary, we have successfully expressed the PKS gene *ChPKS* from *C. higginsianum* in *A. nidulans* and *P. crustosum*. Two new compounds, higginidulans A (**7**) and B (**8**), were identified following heterologous expression in *A. nidulans*. Their formation was confirmed to result from crosstalk of two biosynthetic pathways, i.e. the introduced ChPKS and the cichorine pathway of the host strain *A. nidulans* LO8030. Moreover, the cichorine intermediate **9** was demonstrated to undergo different coupling reactions with the DHN-melanin precursor 1,3,6,8-THN (**1**). Heterologous expression of *ChPKS* in the alternative host system *P. crustosum* JZ52 resulted in the expected formation of the 1,3,6,8-THN (**1**) monomer. Additionally, it was demonstrated that *ChPKS* is capable of replacing the function of the naphthopyrone synthase gene *wA* in *P. crustosum* with regard to spore pigmentation. In addition to providing valuable insight into pathway crosstalk, the findings of this study also highlight the significance of employing alternative host systems for heterologous gene expression, particularly with respect to product modifications by host enzymes or metabolites.

## Electronic supplementary material

Below is the link to the electronic supplementary material.

Supplementary Material 1

## Data Availability

No datasets were generated or analysed during the current study.
