# Supplementary material for: Expression of a Colletotrichum polyketide synthase gene in Aspergillus nidulans leads to unexpected conjugates with a host metabolite
Source: Arch Microbiol. 2025 Feb 6;207(3):52. doi: 10.1007/s00203-025-04258-7 (PMC11802603; doi:10.1007/s00203-025-04258-7)
Supplement: Supplementary file 1 — Supplementary Material 1 [file 203_2025_4258_MOESM1_ESM.pdf]

Supporting Information

Expression of a *Colletotrichum* polyketide synthase gene in *Aspergillus nidulans* leads to unexpected conjugates with host metabolite

David Breyer [‡], Leyao Chen[‡], Jenny Zhou, Zhang-Hai Li, Shu-Ming Li*

Institut für Pharmazeutische Biologie und Biotechnologie, Fachbereich Pharmazie, Philipps-Universität Marburg, Robert-Koch-Straße 4, Marburg 35037, Germany.

[‡] These authors contributed equally

Table of Contents

Supplementary Tables 3

Table S1 Strains used in this study 3

Table S2 Plasmids used in this study 4

Table S3 Primers used in this study 5

Table S4 NMR data of higginidulan A (**7**) 6

Table S5 NMR data of higginidulan B (**8**) 7

Supplementary Figures 8

Fig. S1 PCR verification of transformants 8

Fig. S2 PCR verification of transformants and strategy for ANIA_06448 deletion in *Aspergillus nidulans* DB04 to obtain deletion mutant *Aspergillus nidulans* DB09 9

Fig. S3 LC-MS analysis of a 21-day old culture of *Aspergillus nidulans* DB04 10

Fig. S4 LC-MS analysis of a 21-day old culture of *Aspergillus nidulans* DB09 11

Fig. S5 ^{1}H-NMR spectrum of higginidulan A (**7**) in DMSO-d_6 (500 MHz) 12

Fig. S6 ^{13}C-NMR spectrum of higginidulan A (**7**) in DMSO-d_6 (125 MHz) 13

Fig. S7 HSQC spectrum of higginidulan A (**7**) in DMSO-d_6 14

Fig. S8 HMBC spectrum of higginidulan A (**7**) in DMSO-d_6 15

Fig. S9 NOESY spectrum of higginidulan A (**7**) in DMSO-d_6 16

Fig. S10 ^{1}H-NMR spectrum of higginidulan B (**8**) in acetone-d_6 (500 MHz) 17

Fig. S11 ^{13}C-NMR spectrum of higginidulan B (**8**) in acetone-d_6 (125 MHz) 18

Fig. S12 HSQC spectrum of higginidulan B (**8**) in acetone-d_6 19

Fig. S13 HMBC spectrum of higginidulan B (**8**) in acetone-d_6 20

Fig. S14 ^{1}H-NMR spectrum of 1,3,6,8-tetraacetoxynaphthalene (**11**) in $CDCl_3$ (500 MHz)$_6$ 21

References 22

Supplementary Tables

Table S1 Strains used in this study

Organism	Strain	Genotype	Reference/ Source
Colletotrichum higginsianum	MAFF 305635	wild type	(Voll et al. 2012)
Escherichia coli	DH5α	F– *endA1 glnV44 thi-1 recA1 relA1 gyrA96 deoR nupG purB20* ϕ80d*lacZ*ΔM15 Δ(*lacZYA-argF*)U169, hsdR17(r_K– m_K+), λ–	(Green and Sambrook 2012)
Saccharomyces cerevisiae	HOD114-2B	*MATα ura3-52 his3Δ1 leu2-3112*	(Mojardín et al. 2018)
Aspergillus nidulans	LO8030	*pyroA4, riboB2, pyrG89, nkuA::argB deletion of* sterigmatocystin cluster (*ANIA_07804-ANIA_07825*), emericellamide cluster (*ANIA_02545–ANIA_02549*), asperfuranone cluster (*ANIA_01039–ANIA_01029*), monodictyphenone cluster (*ANIA_10023–ANIA_10021*), terrequinone cluster (*ANIA_08512–ANIA_08520*), austinol cluster part 1 (*ANIA_08379–ANIA_08384*), austinol cluster part 2 (*ANIA_09246–ANIA_09259*), F9775 cluster (*ANIA_07906–ANIA_07915*), asperthecin cluster (*ANIA_06000–ANIA_06002*)	(Chiang et al. 2016)
	SSt01	*wA-PKS*::*PgpdA-AfpyrG* in *Aspergillus nidulans* LO8030	(Stierle and Li 2022)
	DB04	*wA-PKS*::*PgpdA-CH35J_010369-AfpyrG* in *A. nidulans* LO8030	this study
	DB09	ΔANIA_06448::*Afpyro* in *A. nidulans* DB04	this study
Penicillium crustosum	JZ52	Δ*ligD* Δ*traA* Δ*claF* Δ*pcr4401*::*wA-PKS* Δ*pcribo* Δ*pyrG*	(Zhou et al. 2024)
	JZ57	*wA-PKS*::*PgpdA-AfpyrG* in *P. crustosum* JZ52	this study
	JZ58	*wA-PKS*::*PgpdA-CH35J_010369-AfpyrG* in *P. crustosum* JZ52	this study

Table S2 Plasmids used in this study

Plasmid	Description	Reference
pSSt05	Expression vector for the *Aspergillus nidulans* LO8030 strain:<br>amp/URA3, *wA* flanking, *PgpdA, Aspergillus fumigatus pyrG (AfpyrG)*	(Stierle and Li 2022)
pDB04	amp/URA3, *wA* flanking, *PgpdA, CH35J_010369*, *Aspergillus fumigatus pyrG (AfpyrG)*	this study
pYWB1	*URA3, wA* flanking*, A. fumigatus pyroA (afpyro), ampR*	(Janzen et al. 2023)
pZL175	1134 bp 5'-UTR PCR fragment of ANIA_06448 in pYWB1	this study
pZL176	1047 bp 3'-UTR PCR fragment of ANIA_06448 in pYWB1	this study

Table S3 Primers used in this study

Primer	Rev./ For.	Sequence 5' → 3'	Targeted amplification
DB24	Rev.	ACACAACATATTTCGTCAGACACAGAATAAC TCTCGGCTTACTCGCAAACCGCTTCACG	DNA of the 1. CH35J_010369 fragment from *C. higg.* MAFF 305635 to construct pDB04
DB25	For.	CGGCAAGATGGTTGAGACCG	
DB26	Rev.	CGAGGAGACGGGTGCACTCG	DNA of the 2. CH35J_010369 fragment from *C. higg.* MAFF 305635 to construct pDB04
DB27	For.	GACTAACAGCTACCCCGCTTGAGCAGACAT CACCGGCATGGAGCACCGGGAACCTCAAC	
DB34	Rev.	GAAATTAGTAAGTGCCTCTTTGGC	1. partial fragment of pDB04 in *A. nid*. DB04 for transformant verification
SSt81	For.	GCGAGCCTTCCATAGTTACG	
p249_JN015ctrl_01	Rev.	GCACTCTGGAAACGAACTCC	2. partial fragment of pDB04 in *A. nid.* DB04 for transformant verification
DB35	For.	GCCACCTCGATTCTTCTTCAG	
ZL_pZL175_REV	Rev.	TGGTAATATCAGATGGTCTCGAACTGACCT TACATTACAGTTAGAAAGGACTGAACGAC	For cloning of pZL175: amplifying the 1134 bp 5'-UTR of ANIA_06448
ZL_pZL175_FOR	For.	GAATAGTGCTGACTGGTACGATCGCATTTG CTCAGGTCAAGTGCATACAAGAGCTGAAC	
ZL_pZL176_REV	Rev.	GCCCTTTGTCATAGTAAAGTGATTCGCGTC ATGCGGCCGCATGCTGACGACGTGAACAG	For cloning of pZL176: amplifying the 1047 bp 3'-UTR of ANIA_06448
ZL_pZL176_FOR	For.	ATAGACGTCAAGGTACACATCCTACGGAGT TACGATCCTAGTCATCTCGCTTAGAGGAG	
ZL_GT_175_REV	Rev.	ACTTCTGCTCCAGAAGCATAC	For verifying the knockout of ANIA_06448
ZL_GT_175_FOR	For.	ATGAAAGCAGCTGAGACCAAG	
ZL_pyroA_REV	Rev.	AGCTCATAAGGGACCTCAAG	For amplifying the 1134 bp 5'-UTR of ANIA_06448 and 2/3 *afpyro* marker from pZL175
ZL_175_FOR	For.	AGTGCATACAAGAGCTGAAC	
ZL_176_REV	Rev.	ATGCTGACGACGTGAACAG	For amplifying the 1047 bp 3'-UTR of ANIA_06448 and 2/3 *afpyro* marker from pZL176
ZL_pyroA_FOR	For.	CTTCCAACGGTACCAATGG	
JZ37	Rev.	GAGACAGGCCACATCGGTGCTGTATTCCTC	1. partial fragment of pSSt05 in *P. crust.* JZ57 for transformant verification
JZ125	For.	GATCCGTAATACGACTCACTATAGGGCCCG CTTGAGAACATTTGGGTCG	
JZ178	Rev.	GCTCAGAAACGCACACTGG	2. partial fragment of pSSt05 in *P. crust.* JZ57 for transformant verification
JZ36	For.	CTATTGGACGCGGTGCCGACTTTATCATCG	
DB34	Rev.	GAAATTAGTAAGTGCCTCTTTGGC	1. partial fragment of pDB04 in *P. crust.* JZ58 for transformant verification
JZ125	For.	GATCCGTAATACGACTCACTATAGGGCCCG CTTGAGAACATTTGGGTCG	
JZ178	Rev.	GCTCAGAAACGCACACTGG	2. partial fragment of pDB04 in *P. crust.* JZ58 for transformant verification
DB35	For.	GCCACCTCGATTCTTCTTCAG	

Table S4 NMR data of higginidulan A (**7**)

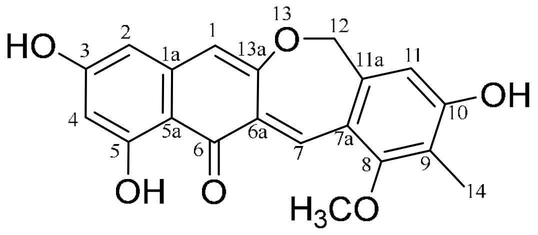

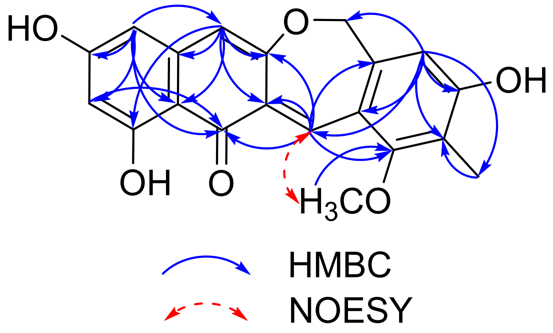

Position	δ_C, type	δ_H, mult (*J* in Hz)	Important HMBC (H→C)
1	104.0, CH	6.10, dd (2.2,1.4)	C-1a, 5, 5a, 6a, 13a
1a	141.0, C		
2	106.3, CH	6.26, d (2.2)	C-1, 3, 5a, 6
3	165.3, C		
4	99.5, CH	6.05, t (2.2)	C-6
5	166.1, C		
5a	106.6, C		
6	186.1, C		
6a	126.5, C		
7	136.8, CH	8.42, br s	C-6, 6a, 8, 11a, 13a
7a	119.3, C		
8	161.8, C		
9	118.0, C		
10	161.8, C		
11	110.8, CH	6.73, s	C-7, 12, 10, 14
11a	140.5, C		
12	71.9, CH_2	4.88, s	
13a	155.5, C		
14	8.9, CH_3	2.10, s	C-9
8-OCH_3	62.3, CH_3	3.76, s	C-8
5-OH		13.77, s	

Table S5 NMR data of higginidulan B (**8**)

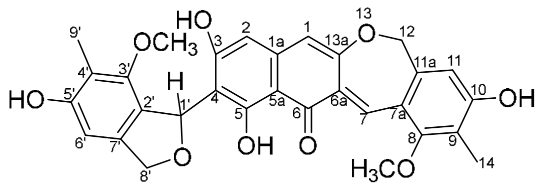

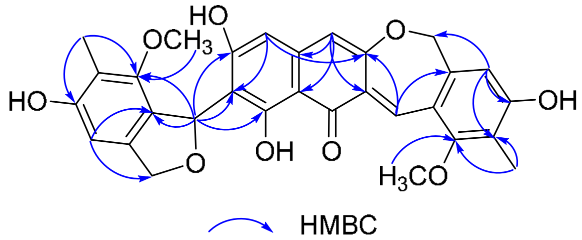

Position	δ_C, type	δ_H, mult (*J* in Hz)	Important HMBC (H→C)
1	104.9, CH	5.96, d (1.2)	
1a	141.3, C		
2	107.1, CH	6.23, s	
3	164.6, C		
4	113.0, C		
5	166.4, C		
5a	108.0, C		
6	188.2, C		
6a	128.4, C		
7	137.9, CH	8.53, d (1.2)	C-11a, 13a
7a	121.2, C		
8	163.2, C		
9	119.3, C		
10	167.3, C		
11	111.6, CH	6.75, s	C-12
11a	141.7, C		
12	73.1, CH_2	4.83, s	
13a	156.9, C		
14	9.2, CH_3	2.14, s	
1'	76.6, CH	6.82, br s	C-4, 2', 3'
2'	124.7, C		
3'	154.6, C		
4'	116.7, C		
5'	157.4, C		
6'	103.4, CH	6.52, s	C-2', 8'
7'	140.8, C		
8'	74.0, CH_2	5.20, br d (11.5); 4.91, br d (11.5)	
9'	9.0, CH_3	1.98, s	C-3', 5'
8-OCH_3	63.0, CH_3	3.79, s	C-8
3'-OCH_3	60.3, CH_3	3.42, s	C-3'
3-OH[a]		8.31, s	
5-OH		14.42, s	
10-OH[a]		9.68, s	
5'-OH[a]		9.18, s	

[a] Assignments are interchangeable

Supplementary Figures

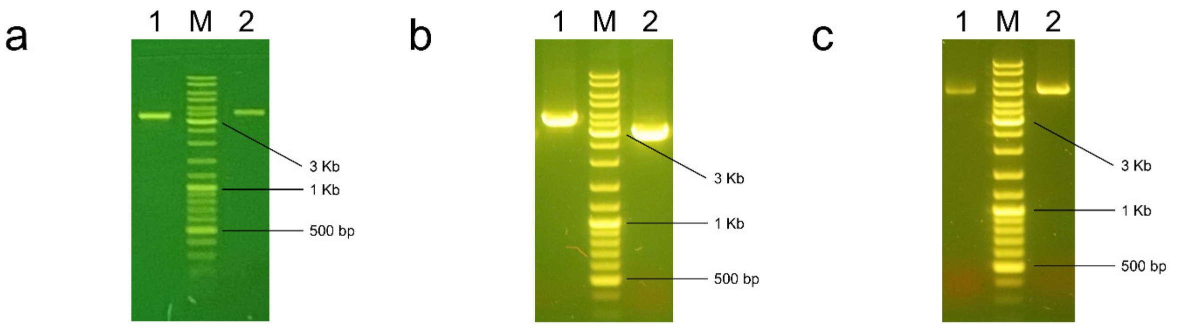

PCR (M: Marker)	Expected band (bp)
	DB04
1: 1. Partial fragment of pDB04	3341
2: 2. Partial fragment of pDB04	4003

PCR (M: Marker)	Expected band (bp)
	JZ57
1: 1. Partial fragment of pSSt05	3677
2: 2. Partial fragment of pSSt05	2948

PCR (M: Marker)	Expected band (bp)
	JZ58
1: 1. Partial fragment of pDB04	4165
2: 2. Partial fragment of pDB04	4989

Fig. S1 (**a**) Verification of the pDB04 insertion into the genome of *Aspergillus nidulans* DB04 by PCR amplification of the 1. partial fragment of pDB04 (1; 3341 bp) and the 2. partial fragment of pDB04 (2; 4003 bp). Primers were selected to bind in the genomic region of *Aspergillus nidulans* as well as in the inserted region. (**b**) Verification of the pSSt05 insertion into the genome of *Penicillium crustosum* JZ57 by PCR amplification of the 1. partial fragment of pSSt05 (3677 bp) and the 2. partial fragment of pSSt05 (2948 bp). (**c**) Verification of the pDB04 insertion into the genome of *Penicillium crustosum* JZ58 by PCR amplification of the 1. partial fragment of pDB04 (4165 bp) and the 2. partial fragment of pDB04 (4989 bp). Primers were selected to bind in the genomic region of *Penicillium crustosum* as well as in the inserted region

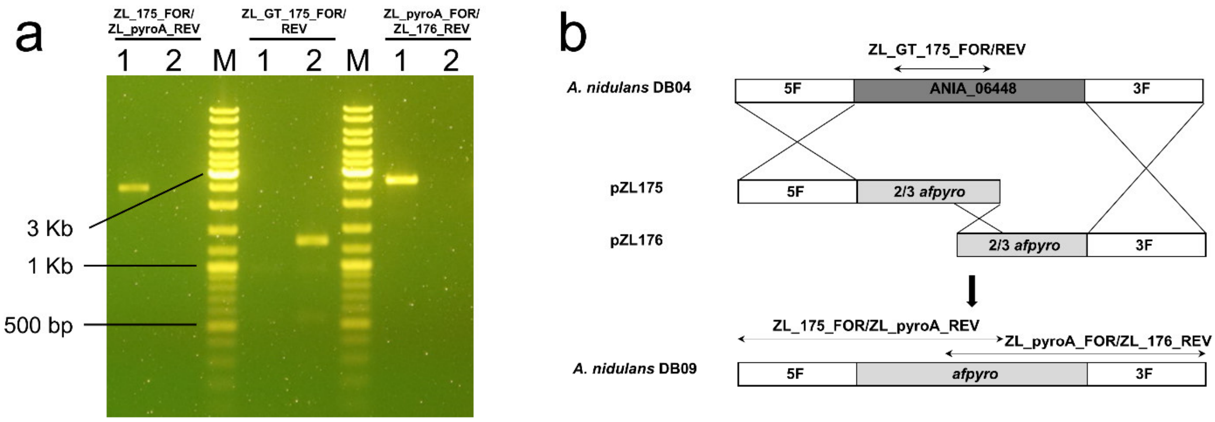

PCR (M: Marker)	Expected band (bp)	
	DB09 (1)	DB04 (2)
ZL_175_FOR/ZL_pyroA_REV	2381	/
ZL_GT_175_FOR/REV	/	1280
ZL_pyroA_FOR/ZL_176_REV	2585	/

Fig. S2 (**a**) Verification of the insertion of *afpyro* (1. partial fragment) replacing ANIA_06448 in *Aspergillus nidulans* DB09 with the primer pair ZL_175_FOR/ZL_pyroA_REV (DB09 (1): 2381 bp; DB04 (2): no band); Verification of the knockout of ANIA_06448 in *Aspergillus nidulans* DB09 and the presence of ANIA_06448 in *Aspergillus nidulans* DB04 with the primer pair ZL_GT_175_FOR/REV (DB09 (1): no band; DB04 (2): 1280 bp); Verification of the insertion of *afpyro* (2. partial fragment) replacing ANIA_06448 in *Aspergillus nidulans* DB09 with the primer pair ZL_pyroA_FOR/ZL_176_REV (DB09 (1): 2585 bp; DB04 (2): no band). (**b**) Strategy for ANIA_06448 deletion in *Aspergillus nidulans* DB04 to obtain deletion mutant *Aspergillus nidulans* DB09

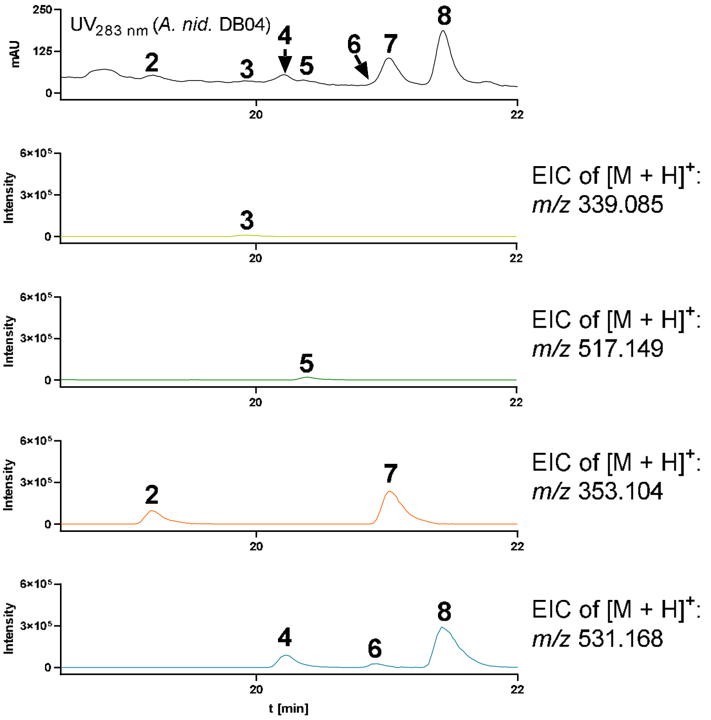

Possible structures for **2**	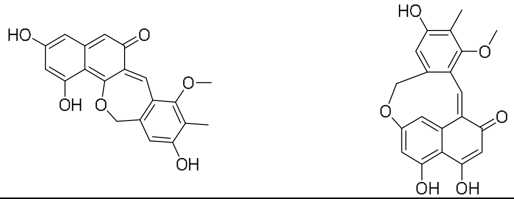
Possible structures for **3**	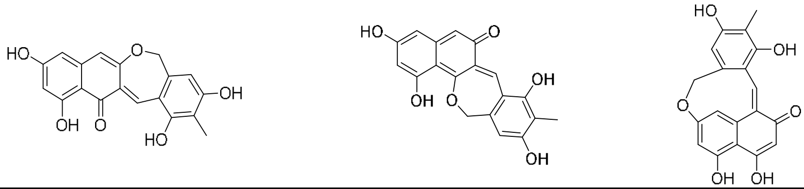
Possible structures for **4** and **6**	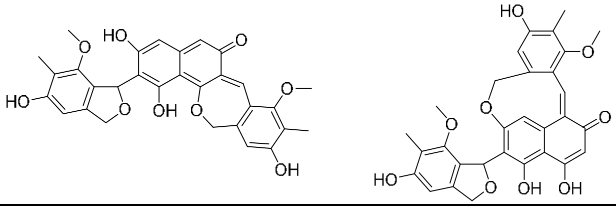
Possible structures for **5**	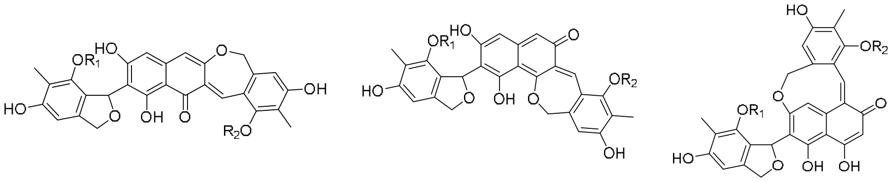<br>R_1 = -CH_3, R_2 = H; R_1 = H, R_2 = -CH_3 (each of the three structures)

Fig. S3 LC-MS analysis of a 21-day old culture of *Aspergillus nidulans* DB04. UV absorptions at 283 nm (black) and EICs of the $[M + H]^+$ ions of products **2** – **8** with a tolerance range of ±0.005 (in color) are illustrated. Possible structures for **2** – **6** are given below the chromatograms. The position of the methoxy group in the structures is based on the possible intermediate **9** of cichorine biosynthetic pathway (Sanchez et al. 2012; Zhao et al. 2023)

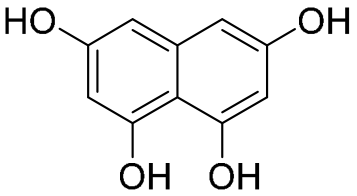

1

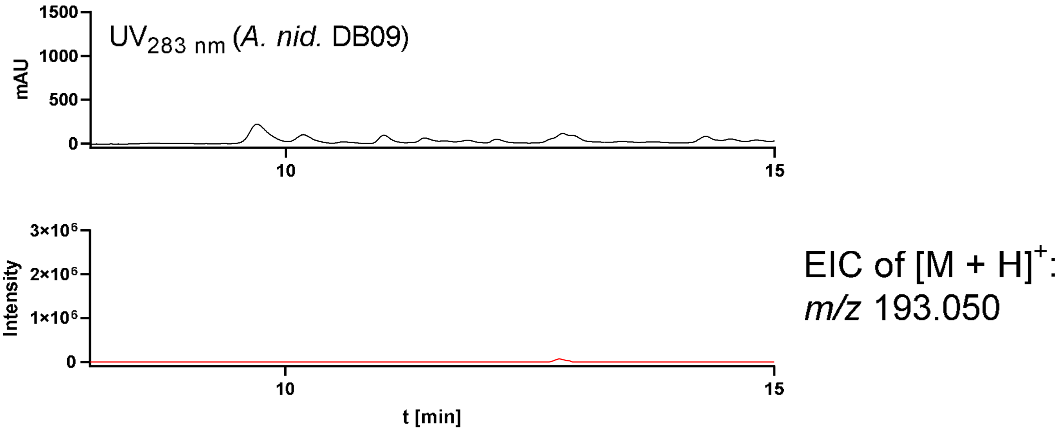

Fig. S4 LC-MS analysis of a 21-day old culture of *Aspergillus nidulans* DB09. UV absorptions at 283 nm (black) and EIC for **1** (in red) with a tolerance range of ±0.005 are illustrated

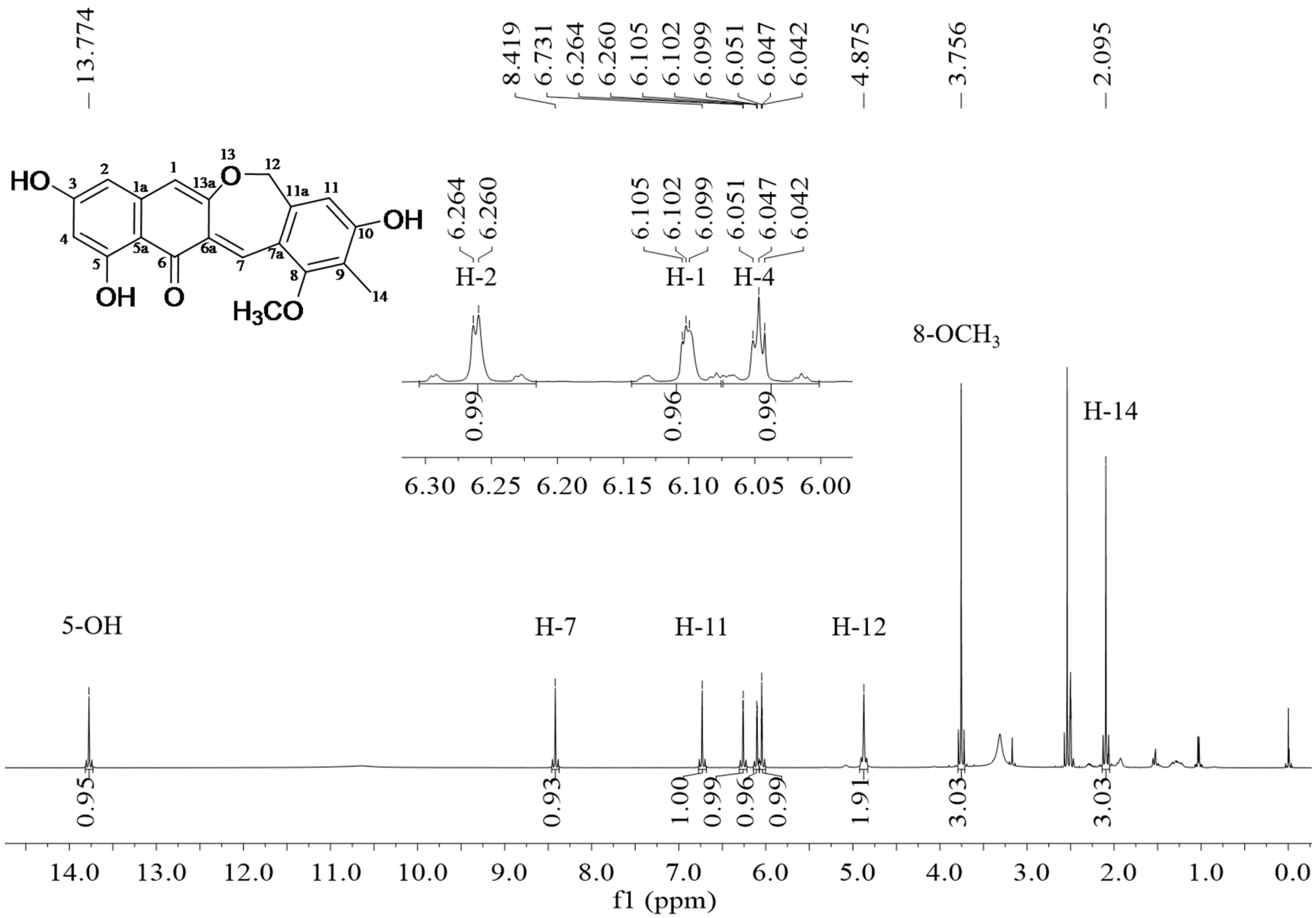

Fig. S5 ^{1}H-NMR spectrum of higginidulan A (**7**) in DMSO-d_6 (500 MHz)

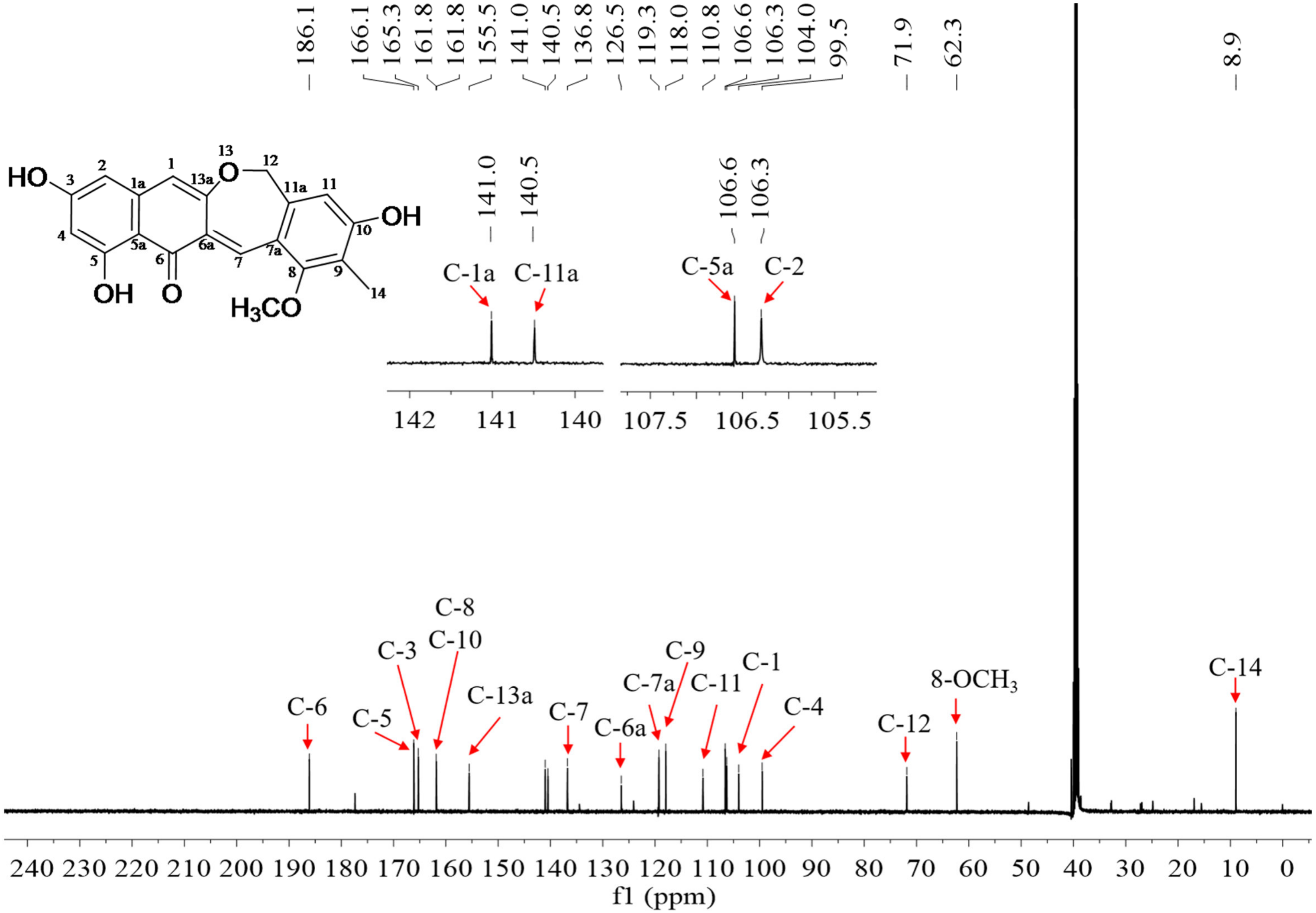

Fig. S6 ^{13}C-NMR spectrum of higginidulan A (**7**) in DMSO-d_6 (125 MHz)

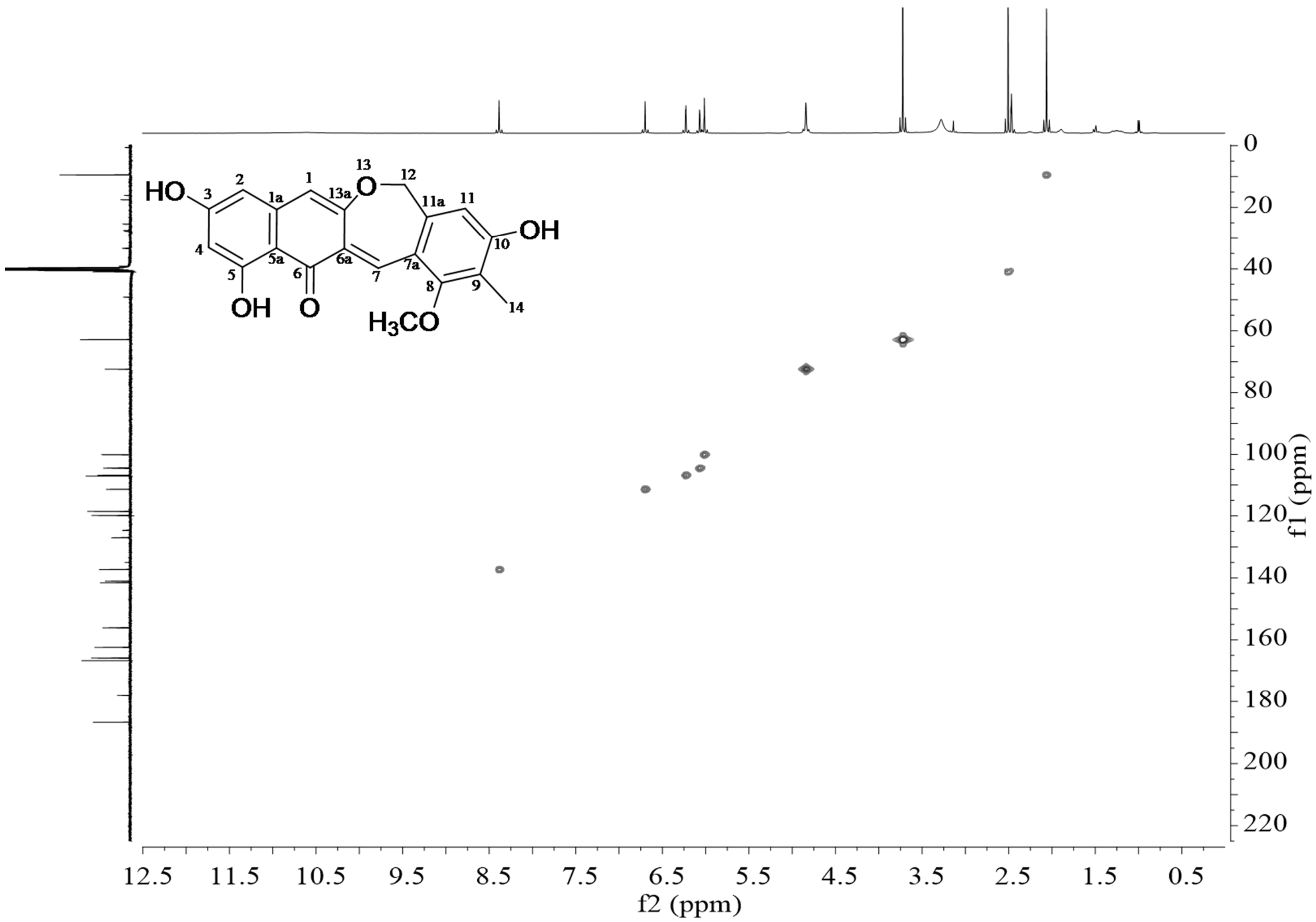

Fig. S7 HSQC spectrum of higginidulan A (**7**) in DMSO-d_6

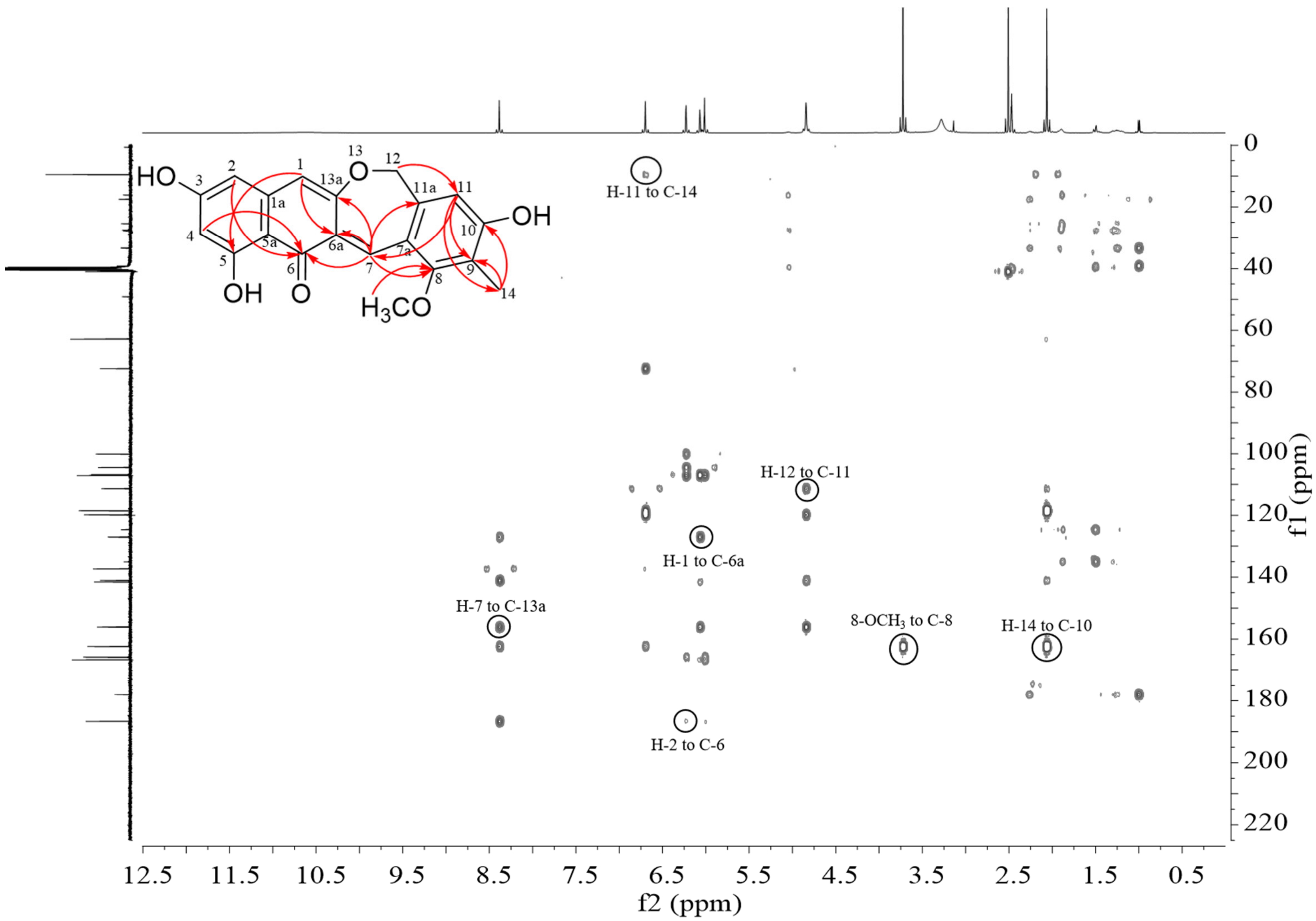

Fig. S8 HMBC spectrum of higginidulan A (**7**) in DMSO-d_6. Observed HMBC correlations are indicated in the structure and only several representatives are marked in the spectrum

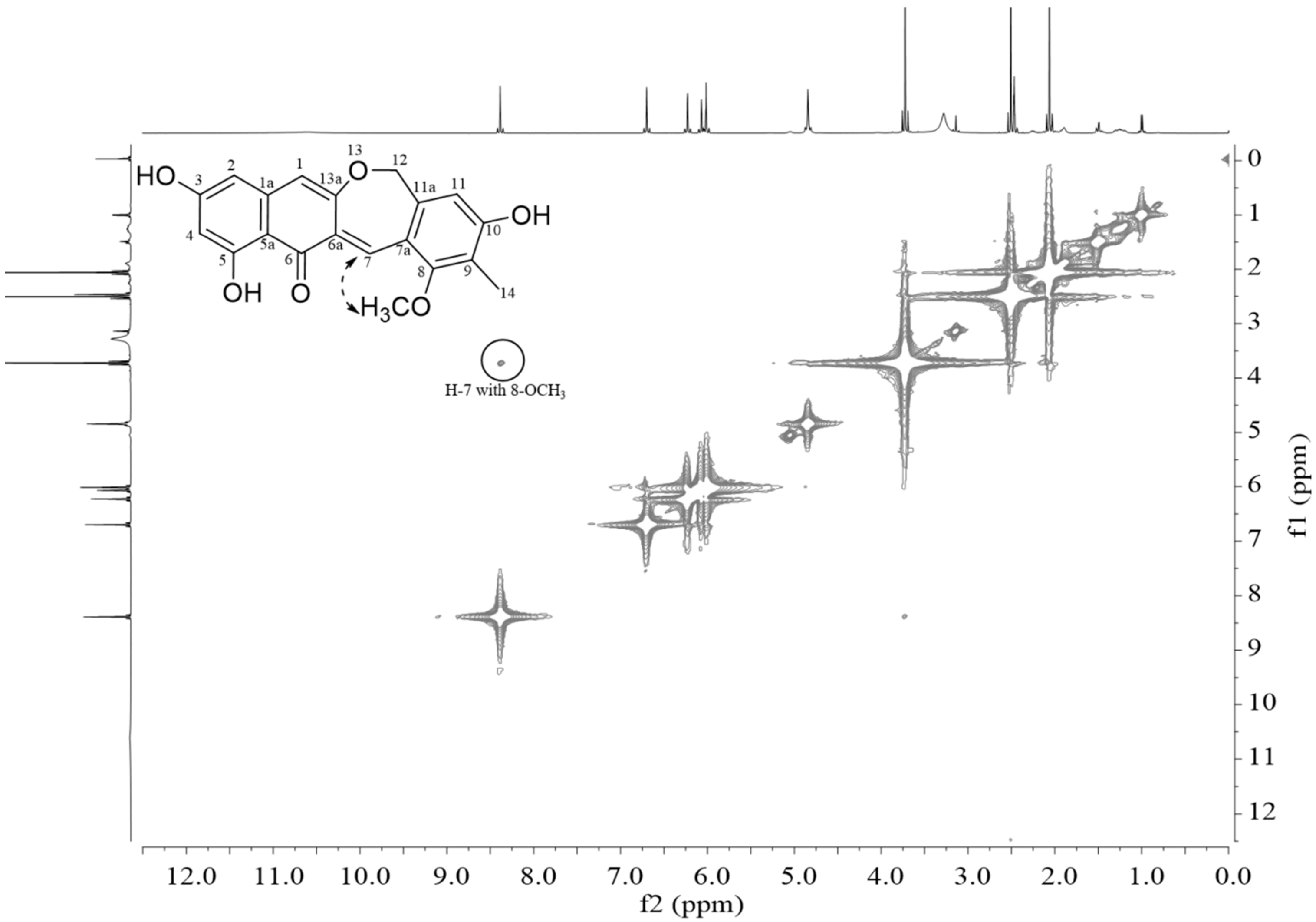

Fig. S9 NOESY spectrum of higginidulan A (**7**) in DMSO-d_6

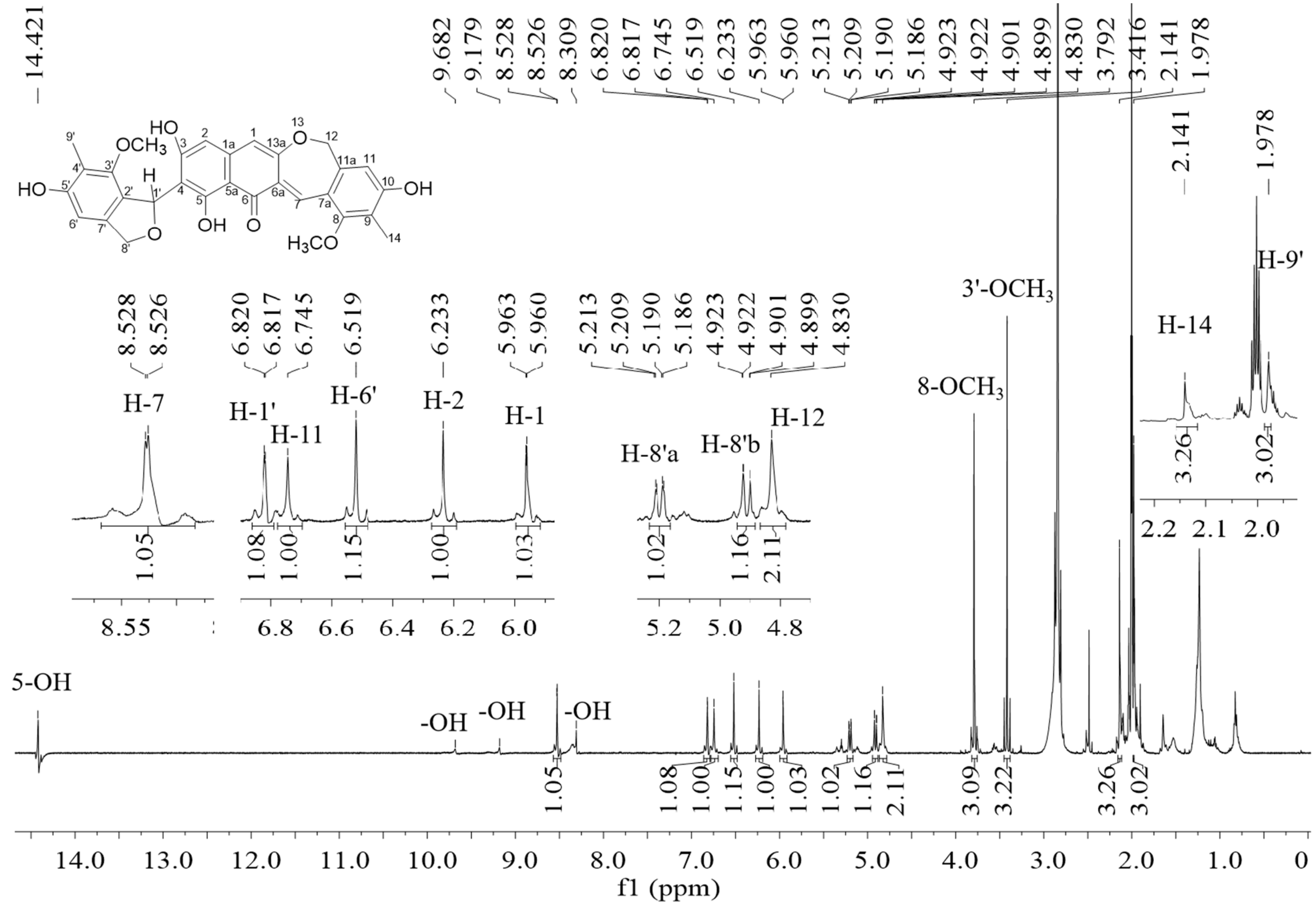

Fig. S10 ^{1}H-NMR spectrum of higginidulan B (**8**) in acetone-d_6 (500 MHz)

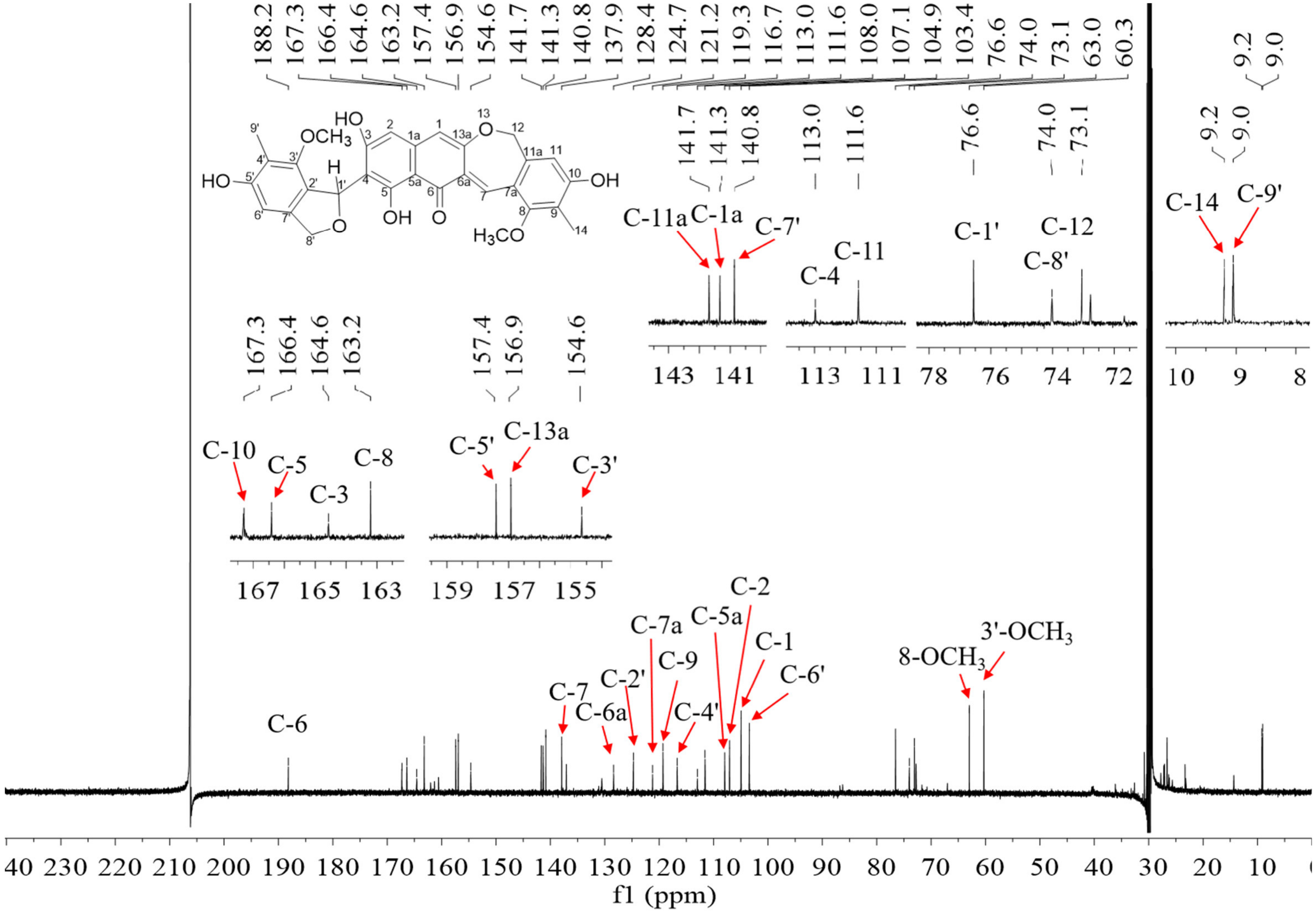

Fig. S11 ^{13}C-NMR spectrum of higginidulan B (**8**) in acetone-d_6 (125 MHz)

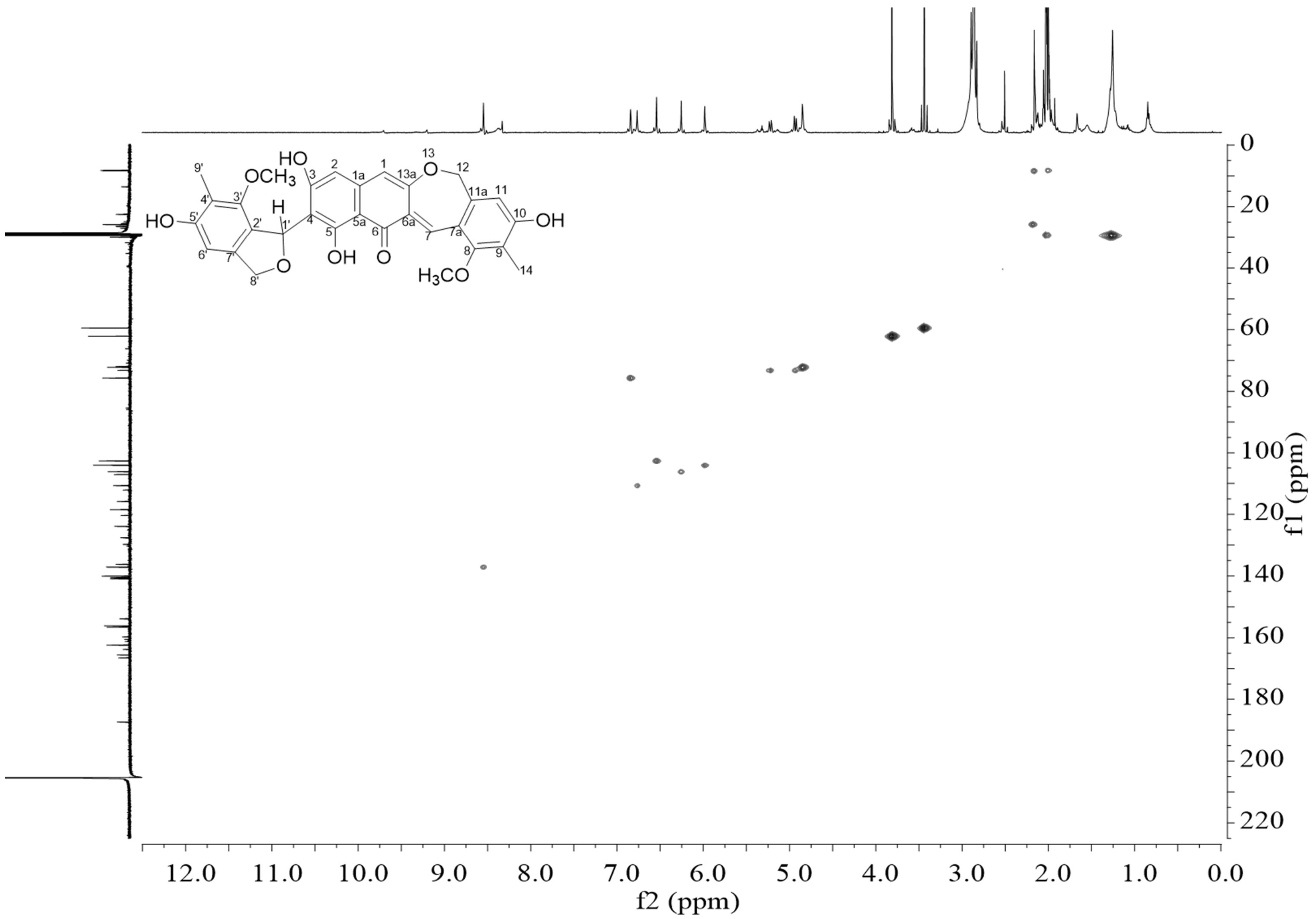

Fig. S12 HSQC spectrum of higginidulan B (**8**) in acetone-d_6

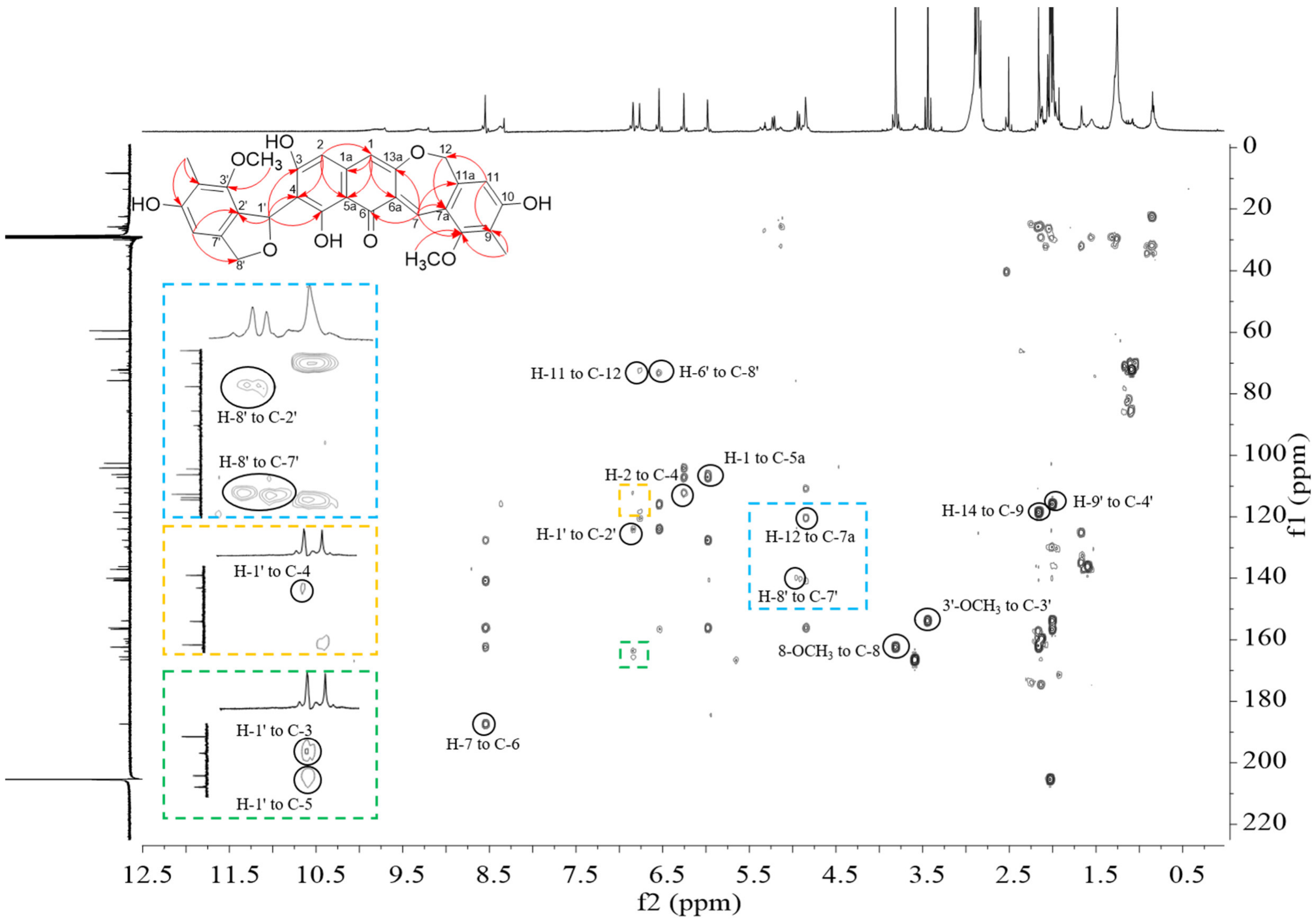

Fig. S13 HMBC spectrum of higginidulan B (**8**) in acetone-d_6. Observed HMBC correlations are indicated in the structure and only several representatives are marked in the spectrum. Three framed regions are enlarged.

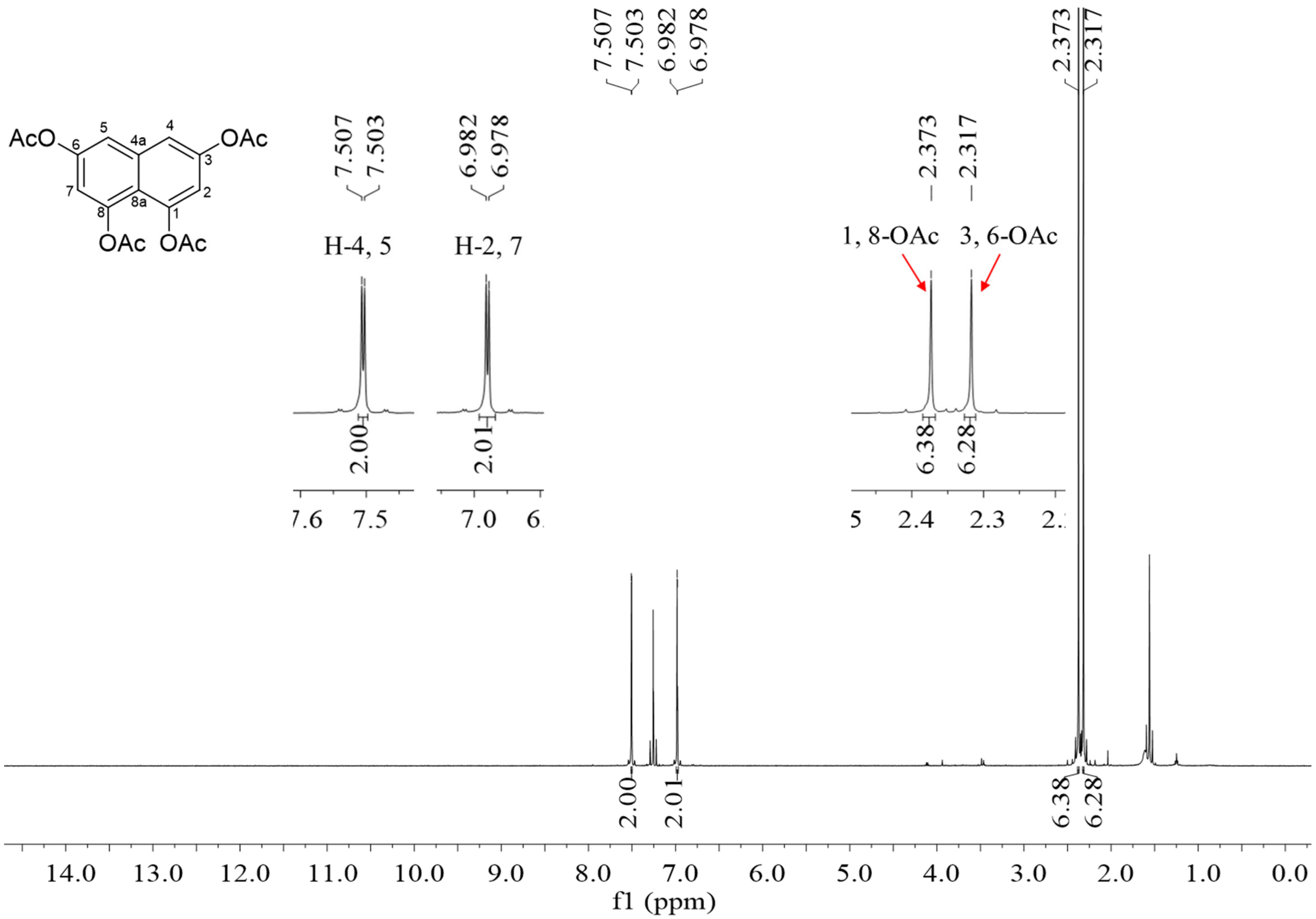

Fig. S14 ^{1}H-NMR spectrum of 1,3,6,8-tetraacetoxynaphthalene (**11**) in $CDCl_3$ (500 MHz)[6]

References

Chiang YM, Ahuja M, Oakley CE, Entwistle R, Asokan A, Zutz C, Wang CC, Oakley BR (2016) Development of genetic dereplication strains in *Aspergillus nidulans* results in the discovery of aspercryptin. Angew Chem Int Ed 55:1662–1665. https://doi.org/10.1002/anie.201507097

Green MR, Sambrook J (2012) Molecular cloning: a laboratory manual, 4th. Cold Spring Harbor Laboratory Press, Cold Spring Harbor, New York

Janzen DJ, Zhou J, Li S-M (2023) Biosynthesis of *p*-terphenyls in *Aspergillus ustus* implies enzymatic reductive dehydration and spontaneous dibenzofuran formation. Org Lett 25:6311–6316. https://doi.org/10.1021/acs.orglett.3c02234

Mojardín L, Vega M, Moreno F, Schmitz HP, Heinisch JJ, Rodicio R (2018) Lack of the NAD^+-dependent glycerol 3-phosphate dehydrogenase impairs the function of transcription factors Sip4 and Cat8 required for ethanol utilization in *Kluyveromyces lactis*. Fungal Genet Biol 111:16–29. https://doi.org/10.1016/j.fgb.2017.11.006

Sanchez JF, Entwistle R, Corcoran D, Oakley BR, Wang CCC (2012) Identification and molecular genetic analysis of the cichorine gene cluster in *Aspergillus nidulans*. MedChemComm 3:997–1002. https://doi.org/10.1039/C2MD20055D

Stierle SA, Li S-M (2022) Biosynthesis of xylariolide D in *Penicillium crustosum* implies a chain branching reaction catalyzed by a highly reducing polyketide synthase. J. Fungi (Basel) 8:493. https://doi.org/10.3390/jof8050493

Voll LM, Zell MB, Engelsdorf T, Saur A, Wheeler MG, Drincovich MF, Weber APM, Maurino VG (2012) Loss of cytosolic NADP-malic enzyme 2 in *Arabidopsis thaliana* is associated with enhanced susceptibility to *Colletotrichum higginsianum*. New Phytol. 195:189–202. https://doi.org/10.1111/j.1469-8137.2012.04129.x

Zhao F, Sun C, Liu Z, Cabrera A, Escobar M, Huang S, Yuan Q, Nie Q, Luo KL, Lin A, Vanegas JA, Zhu T, Hilton IB, Gao X (2023) Multiplex base-editing enables combinatorial epigenetic regulation for genome mining of fungal natural products. J Am Chem Soc 145:413–421. https://doi.org/10.1021/jacs.2c10211

Zhou J, Chen XL, Li SM (2024) Construction of an expression platform for fungal secondary metabolite biosynthesis in *Penicillium crustosum*. Appl Microbiol Biotechnol 108:427. https://doi.org/10.1007/s00253-024-13259-3
